# Genetically predicted sex hormone levels and health outcomes: phenome-wide Mendelian randomization investigation

**DOI:** 10.1093/ije/dyac036

**Published:** 2022-02-26

**Authors:** Shuai Yuan, Lijuan Wang, Jing Sun, Lili Yu, Xuan Zhou, Jie Yang, Yimin Zhu, Dipender Gill, Stephen Burgess, Joshua C Denny, Susanna C Larsson, Evropi Theodoratou, Xue Li

**Affiliations:** Department of Big Data in Health Science, Center of Clinical Big Data and Analytics of the Second Affiliated Hospital, Zhejiang University School of Medicine, Hangzhou, China; Unit of Cardiovascular and Nutritional Epidemiology, Institute of Environmental Medicine, Karolinska Institutet, Stockholm, Sweden; Department of Big Data in Health Science, Center of Clinical Big Data and Analytics of the Second Affiliated Hospital, Zhejiang University School of Medicine, Hangzhou, China; Department of Big Data in Health Science, Center of Clinical Big Data and Analytics of the Second Affiliated Hospital, Zhejiang University School of Medicine, Hangzhou, China; Department of Big Data in Health Science, Center of Clinical Big Data and Analytics of the Second Affiliated Hospital, Zhejiang University School of Medicine, Hangzhou, China; Department of Big Data in Health Science, Center of Clinical Big Data and Analytics of the Second Affiliated Hospital, Zhejiang University School of Medicine, Hangzhou, China; Department of Big Data in Health Science, Center of Clinical Big Data and Analytics of the Second Affiliated Hospital, Zhejiang University School of Medicine, Hangzhou, China; Department of Big Data in Health Science, Center of Clinical Big Data and Analytics of the Second Affiliated Hospital, Zhejiang University School of Medicine, Hangzhou, China; Department of Epidemiology and Biostatistics, School of Public Health, Imperial College London, London, UK; MRC Biostatistics Unit, Cambridge Institute of Public Health, Cambridge, UK; Department of Biomedical Informatics, Vanderbilt University Medical Center, Nashville, TN, USA; Unit of Cardiovascular and Nutritional Epidemiology, Institute of Environmental Medicine, Karolinska Institutet, Stockholm, Sweden; Unit of Medical Epidemiology, Department of Surgical Sciences, Uppsala University, Uppsala, Sweden; Centre for Global Health, Usher Institute, University of Edinburgh, Edinburgh, UK; Cancer Research UK Edinburgh Centre, Medical Research Council Institute of Genetics and Cancer, University of Edinburgh, Edinburgh, UK; Department of Big Data in Health Science, Center of Clinical Big Data and Analytics of the Second Affiliated Hospital, Zhejiang University School of Medicine, Hangzhou, China; Centre for Global Health, Usher Institute, University of Edinburgh, Edinburgh, UK

**Keywords:** Cardiovascular disea, se, o, estradiol, Mendelian randomization, testosterone, sex hormone-binding globulin

## Abstract

**Background:**

Sex hormone-binding globulin (SHBG), testosterone and oestradiol have been associated with many diseases in observational studies; however, the causality of associations remains unestablished.

**Methods:**

A phenome-wide Mendelian randomization (MR) association study was performed to explore disease outcomes associated with genetically proxied circulating SHBG, testosterone and oestradiol levels by using updated genetic instruments in 339 197 unrelated White British individuals (54% female) in the UK Biobank. Two-sample MR analyses with data from large genetic studies were conducted to replicate identified associations in phenome-wide MR analyses. Multivariable MR analyses were performed to investigate mediation effects of hormone-related biomarkers in observed associations with diseases.

**Results:**

Phenome-wide MR analyses examined associations of genetically predicted SHBG, testosterone and oestradiol levels with 1211 disease outcomes, and identified 28 and 13 distinct phenotypes associated with genetically predicted SHBG and testosterone, respectively; 22 out of 28 associations for SHBG and 10 out of 13 associations for testosterone were replicated in two-sample MR analyses. Higher genetically predicted SHBG levels were associated with a reduced risk of hypertension, type 2 diabetes, diabetic complications, coronary atherosclerotic outcomes, gout and benign and malignant neoplasm of uterus, but an increased risk of varicose veins and fracture (mainly in females). Higher genetically predicted testosterone levels were associated with a lower risk of type 2 diabetes, coronary atherosclerotic outcomes, gout and coeliac disease mainly in males, but an increased risk of cholelithiasis in females.

**Conclusions:**

These findings suggest that sex hormones may causally affect risk of several health outcomes.

Key Messages

- Phenome-wide Mendelian randomization analyses examined associations of genetically predicted sex hormone-binding globulin (SHBG), testosterone and oestradiol levels with 1211 disease outcomes, and identified 28 and 13 distinct phenotypes associated with genetically predicted SHBG and testosterone, respectively.
- Of 28 associations for SHBG, 22 were replicated in two-sample Mendelian randomization analyses, as were 10 of 13 associations for testosterone.
- Three phenotypes were associated with genetically predicted high oestradiol levels in males in phenome-wide Mendelian randomization analyses but not in two-sample Mendelian randomization analyses.
- An improved blood lipid profile appeared to largely mediate the protective effects of higher genetically predicted SHBG and testosterone levels on coronary atherosclerotic diseases.

## Introduction

Sex hormones, including androgens and oestrogens, play important roles in controlling secondary sex characteristics. Sex hormone-binding globulin (SHBG), a circulating glycoprotein binding to androgens and oestrogens, is a major transporter and putative regulator of sex hormones and enhances and inhibits hormonal influences.

Sex hormones and SHBG have been associated with a wide range of health outcomes. Most, but not all, observational studies found that high levels of endogenous testosterone were associated with a decreased risk of atherosclerosis,^1^^,^^2^ type 2 diabetes,^1^^,^^2^ cardiovascular diseases^1^^,^^2^ and osteoporosis,^3^ but an increased risk of thrombosis^4^ and certain cancers.^5^^,^^6^ Likewise, observational studies on SHBG revealed relatively consistent evidence on the associations with diabetes,^7^ prostate cancer^5^ and fracture.^8^ However, the effects of endogenous oestradiol have not been thoroughly examined. Randomized clinical trials have attempted to assess the benefits and adversity of testosterone and oestrogen therapies.^9–12^ Even though certain outcomes have been identified to be associated with these treatments,^9–12^ the long-term influences and side effects of sex hormone therapy cannot be satisfactorily determined, due to logistic and financial challenges.^10^

Phenome-wide Mendelian randomization (MR) analysis has been proposed as a hypothesis-searching approach to comprehensively examine causality between exposure and a wide range of disease outcomes.^13^ The method can minimize residual confounding and diminish reverse causality. Previous phenome-wide MR and two-sample MR studies have explored the associations of SHBG and testosterone,^14^^,^^15^ including associations of SHBG with lipid metabolism,^15^ cardiovascular disease,^16^ kidney function^17^ and endometrial cancer,^18^ and associations of testosterone with lipid metabolism^14^ and cardiovascular disease.^16^^,^^19^ However, some evidence is conflicting, especially for the associations with cardiovascular disease.^14–16^^,^^19^ Additionally, some previous studies may be confined by the small number of genetic variants and lack of sex-specific effect.^14^^,^^15^ Thus, we performed updated phenome-wide MR and subsequent two-sample MR studies to examine health outcomes associated with SHBG, testosterone and oestradiol levels across the phenome.

## Methods

### Study design

**Figure 1:**
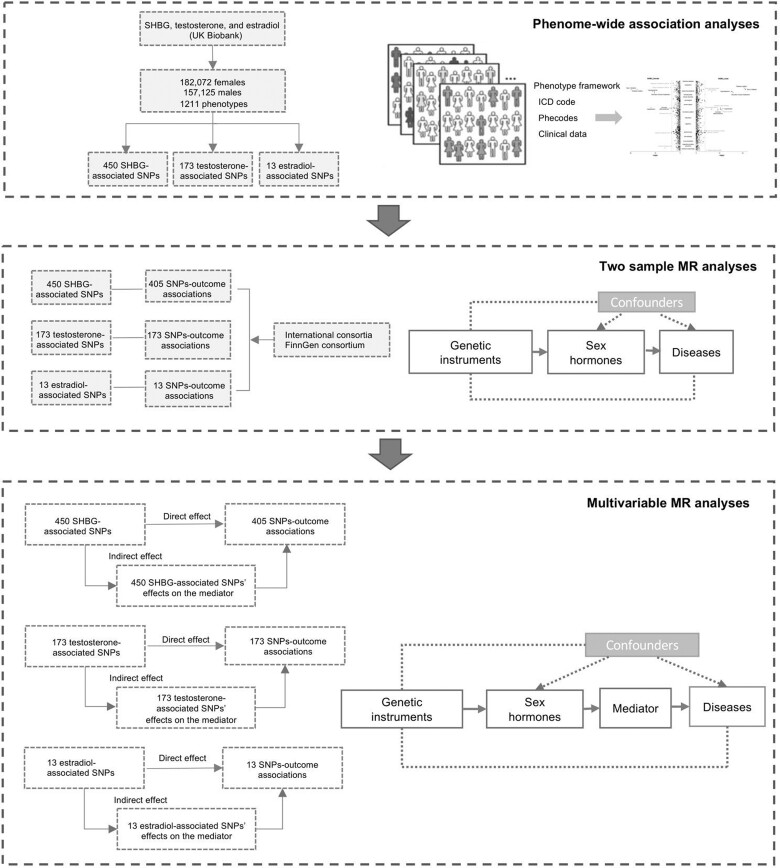
Study design overview. MR, Mendelian randomization; SNPs, single nucleotide polymorphisms; SHBG, sex hormone-binding globulin

Figure 1 shows the study design and the rationale of MR analysis. We first performed phenome-wide investigation to explore all possible outcomes associated with genetically predicted SHBG, testosterone and oestradiol levels in the UK Biobank study. For associations that survived the multiple testing correction, we further performed two-sample MR analyses for replication. Multivariable MR analyses were conducted to estimate the mediation of hormone-associated biomarkers in the associations between hormones and diseases.

### Genetic instrument selection

Single nucleotide polymorphisms (SNPs) associated with SHBG (females and males), testosterone (females and males) and oestradiol (males) levels were identified from genome-wide association analyses in up to 425 097 participants.^20^ After excluding SNPs with linkage disequilibrium (*r^2^* >0.01), 450, 173 and 13 SNPs were used as instrumental variables for SHBG, testosterone and oestradiol, respectively. The same instruments were used in analyses in females and males combined and by sex. Detailed information on SNPs used and genome-wide association analyses is presented in Supplementary Tables S1 and S2 (available as Supplementary data at *IJE* online).

### Phenome-wide MR analysis

The phenome-wide MR analysis was conducted in the UK Biobank study. The UK Biobank study is a large-scale prospective cohort study including 500 000 individuals aged 40–69 years in 2006–10. The study collected data on genotype and a wide range of health outcomes from national medical records. A detailed description on genotype and phenotype data and quality control in UK Biobank is presented in Supplementary Methods (available as Supplementary data at *IJE* online). The PheCODE schema was used to define phenotypes, based on an integrative application of 10 750 unique ICD-10 codes and 3113 ICD-9 codes.^21^ In this study, we confined the studied population to a subgroup of unrelated White British individuals, to minimize population structure bias.

### Two-sample MR analysis

We replicated associations identified in phenome-wide MR analyses using the two-sample MR analysis method. Summary-level data on the associations of hormone-associated SNPs with identified outcomes in phenome-wide MR analyses were obtained from genetic consortia^22–31^ and the FinnGen consortium^32^ (Supplementary Table S3, available as Supplementary data at *IJE* online).

### Multivariable MR analysis

Biomarkers or traits selected for the multivariable MR analysis were based on the results of the phenome-wide MR and two-sample MR analyses. In particular, we first assessed the associations of genetically predicted SHBG and hormones with the intermediate traits or biomarkers (i.e. blood pressures, lipids, glycaemic traits and uric acid) of these disease outcomes, to pinpoint traits or biomarkers possibly associated with SHBG and hormones. We then adjusted for these associated biomarkers or traits in the multivariable MR analysis, in which the same sets of genetic instruments for sex hormones were used and their genetic associations with these biomarkers or traits were adjusted in the MR analysis. The corresponding genome-wide association summary-level data sources (e.g. International Consortium of Blood Pressure, Global Lipids Genetics Consortium) are summarized in Supplementary Table S3.

### Statistical analysis

In the phenome-wide analysis, selected genetic instruments (i.e. SNPs) associated with SHBG, testosterone and oestradiol were used to construct weighted genetic risk scores. The weighted genetic risk score was created by summing the number of hormone-increasing alleles for each SNP weighted by effect size on hormone levels and then adding this weighted score for all used SNPs. In this analysis, we only included outcomes with more than 120 cases, and this minimum number of cases was determined based on an estimation of study power for phenome-wide MR analysis, as shown in Supplementary Figure S1 (available as Supplementary data at *IJE* online). The associations of genetically predicted hormone levels with 1211 outcomes were calculated by logistic regression models with adjustment for age, sex, body mass index (BMI), assessment centre and the first 10 principal components. We stratified the analyses by sex for SHBG and testosterone. The analysis for oestradiol was conducted only in males, given that corresponding instruments were obtained only in males.^20^ We applied a false discovery rate (FDR) correction using the method by Benjamini-Hochberg to account for multiple comparisons in phenome-wide analysis.^33^

In two-sample MR analysis, the inverse variance weighted method with multiplicative random effects was used as the main statistical method and supplemented with two sensitivity analyses, including the weighted median^34^ and MR-Egger analyses.^35^ We used multivariable MR analysis to examine the mediation effects of hormone-associated biomarkers in the associations between hormones and outcomes.^36^ Network MR method was used to estimate the proportion of the total effect of the hormone on each outcome which is mediated through the hormone-associated biomarker.^37^ Cochrane’s Q value was used to assess the heterogeneity in estimates of SNPs for each association. The *P*-value for intercept in MR-Egger analysis was used to assess the horizontal pleiotropy (*P *<0.05). Two-sample MR associations in different sources were combined using a fixed-effect meta-analysis, and *P *<0.05 was used as significance threshold in the MR analyses for replication.

Phenome-wide MR analysis was conducted using an R package by Carroll *et al*.,^38^ two-sample MR analyses were performed using the TwoSampleMR package^39^ and multivariable and network MR analyses were conducted using MendelianRandomziation package^40^ in R Software 4.0.2.

## Results

### Phenome-wide MR analysis

A total of 339 197 unrelated White British individuals (182 072 females and 157 125 males) was included in this phenome-wide MR analysis. The mean age of the studied population was 56.9 (SD: 8.0) years at the time of recruitment (Supplementary Table S4, available as Supplementary data at *IJE* online). The mean value of the weighted genetic risk score among the study population was 0.11 (SD: 0.15) for SHBG, 0.09 (SD: 0.11) for testosterone and 0.01 (SD: 0.02) for oestradiol, which is equivalent to 0.11 nmol/L of serum SHBG levels, 0.09 nmol/L of serum testosterone levels and 0.01 nmol/L of serum oestradiol levels, respectively.

**Figure 2:**
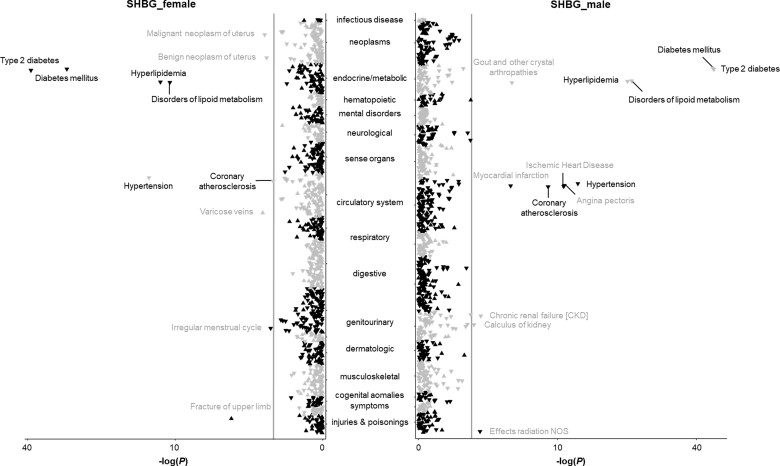
Associations of genetically predicted sex hormone-binding globulin (SHBG) levels with clinical outcomes and biomarkers in the phenome-wide association analysis in the UK Biobank. NOS indicates not other specified. The y-axes correspond to the logarithms of the *P*-values derived from the phenome-wide association analyses. The grey lines correspond to the statistical significance level (false discovery rate <0.05). Associations surviving the significance criteria are labelled by name. Associations that are found in females and males combined are labelled in black, whereas associations that are revealed in females or males are labelled in grey. The triangle facing up represents a positive association, otherwise an inverse association

After removal of outcomes with <120 cases, this phenome-wide MR analysis was performed for 1211 phenotypes classified into 18 broadly related disease categories (Supplementary Table S5, available as Supplementary data at *IJE* online). After accounting for multiple testing, 28 phenotypes were identified to be associated with genetically predicted high serum SHBG levels in all participants (Supplementary Table S6, available as Supplementary data at *IJE* online). Respectively, 18 and 20 associations were observed in the analysis in females and males (Figure 2; and Supplementary Table S6, available as Supplementary data at *IJE* online). These phenotypes present 10 disease outcomes (type 2 diabetes and its complications, irregular menstrual cycle, endometrial cancer, lipid metabolism disorder, hypertension, coronary atherosclerosis, uterine leiomyoma, varicose veins and fracture) in females and 12 disease outcomes (type 2 diabetes and its complications, gout, angina pectoris, lipid metabolism disorder, kidney stones, effects of radiation not other specified, chronic kidney disease, myocardial infarction, coronary atherosclerosis, ischaemic heart disease, and hypertension) in males.

**Figure 3:**
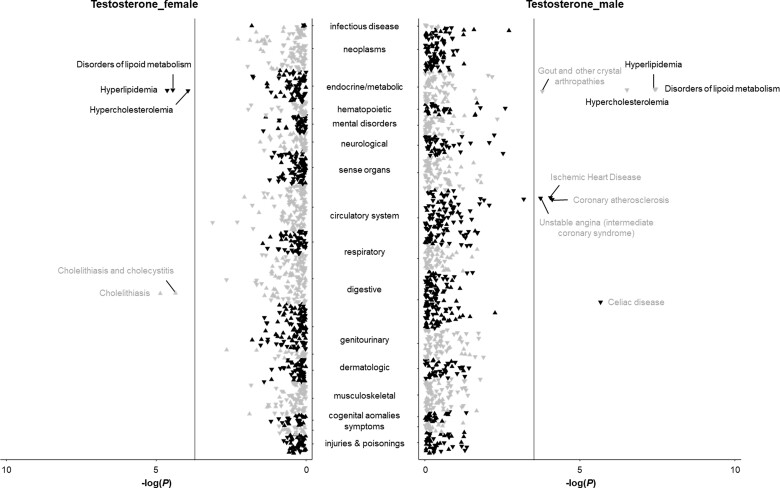
Associations of genetically predicted testosterone levels with clinical outcomes and biomarkers in the phenome-wide association analysis in the UK Biobank. The y-axes correspond to the logarithms of the *P*-values derived from the phenome-wide association analyses. The grey lines correspond to the statistical significance level (false discovery rate <0.05). Associations surviving the significance criteria are labelled by name. Associations that are found in females and males combined are labelled in black, whereas associations that are revealed in females or males are labelled in grey. The triangle facing up represents a positive association, otherwise an inverse association

Results for testosterone are presented in Figure 3 and in Supplementary Table S7 (available as Supplementary data at *IJE* online); 16, five and 10 phenotypes were identified to be associated with genetically predicted high serum testosterone levels in the analyses in females and males combined, females, and males, respectively. Genetically predicted testosterone levels were associated with disorders of lipid metabolism in both females and males. The associations with coeliac disease, gout, unstable angina, coronary atherosclerosis, type 2 diabetes, and ischaemic heart disease were mainly observed in males, and an association with cholelithiasis was observed in females (Supplementary Table S7). A few phenotypes, including disorders of lipid metabolism, polyarteritis nodosa and peripheral vascular disease, were associated with genetically predicted high oestradiol levels in males (Supplementary Table S8 and Supplementary Figure S2, available as Supplementary data at *IJE* online).

### Two-sample MR analysis

In the two-sample MR analyses, 22 of 28 associations for SHBG and 10 of 13 associations for testosterone were replicated. Higher genetically predicted SHBG and testosterone levels were associated with increased levels of high-density lipoprotein cholesterol and lower levels of triglycerides (Table 1). Higher genetically predicted SHBG levels were additionally associated with lower blood pressure and uric acid (Table 1).

**Table 1.** Associations of genetically predicted sex hormone-binding globulin and testosterone levels with biomarkers in two-sample Mendelian randomization analysis

Outcome	Source	Unit	Sample size	Change (95% CI)	*P*
**Sex hormone-binding globulin**					
Total cholesterol	GLGC	SD	187 365	−0.10 (−0.21, 0.02)	0.096
Low-density lipoprotein cholesterol	GLGC	SD	173 082	−0.07 (−0.17, 0.04)	0.216
High-density lipoprotein cholesterol	GLGC	SD	187 167	0.43 (0.32, 0.53)	<0.001
Triglycerides	GLGC	SD	177 861	−0.61 (−0.73, −0.49)	<0.001
Systolic blood pressure	ICBP	mmHg	757 601	−2.72 (−3.60, −1.85)	<0.001
Diastolic blood pressure	ICBP	mmHg	757 601	−1.25 (−1.77, −0.73)	<0.001
Uric acid	CKD Gen consortium	mg/dL	288 649	−0.21 (−0.29, −0.13)	<0.001
Fasting glucose	PAGE study	SD	13 556	−0.08 (−0.19, 0.03)	0.168
**Testosterone**					
Total cholesterol	GLGC	SD	187 365	−0.22 (−0.38, −0.06)	0.007
Low-density lipoprotein cholesterol	GLGC	SD	173 082	−0.07 (−0.22, 0.07)	0.300
High-density lipoprotein cholesterol	GLGC	SD	187 167	0.17 (0.02, 0.32)	0.023
Triglycerides	GLGC	SD	177 861	−0.71 (−0.96, −0.46)	<0.001
Uric acid	CKD Gen consortium	mg/dL	288 649	−0.13 (−0.29, 0.03)	0.114

CI, confidence interval; CKD Gen, Chronic Kidney Disease Genetics; GLGC, Global Lipids Genetics Consortium; ICBP, International Consortium for Blood Pressure; PAGE, Population Architecture using Genomics and Epidemiology; SD, standard deviation.

**Figure 4:**
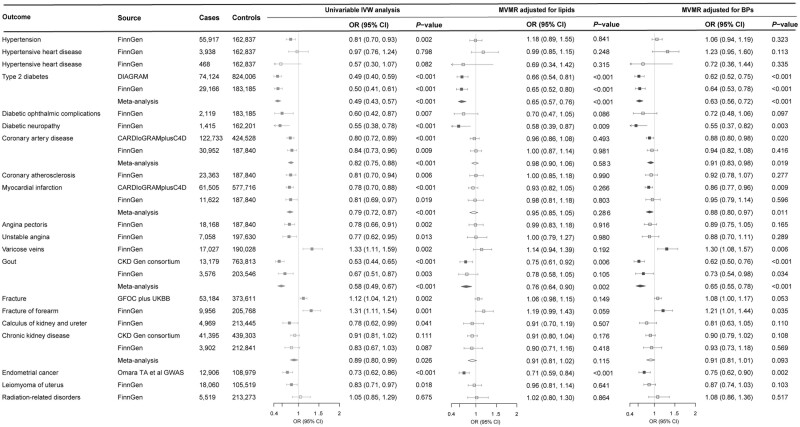
Associations of genetically predicted sex hormone-binding globulin levels with diseases in univariable and multivariable Mendelian randomization analysis. BPs, blood pressures; CARDIoGRAMplusC4D, Coronary ARtery DIsease Genome wide Replication and Meta-analysis (CARDIoGRAM) plus The Coronary Artery Disease (C4D) Genetics; CI, confidence interval; CKD Gen, Chronic Kidney Disease Genetics; DIAGRAM, DIAbetes Genetics Replication And Meta-analysis; GFOC, GEnetic Factors for OSteoporosis Consortium; GWAS, genome-wide association study; IVW, inverse variance weighted; MVMR, multivariable Mendelian randomization; OR, odds ratio; UKBB, UK Biobank. MVMR-adjusted lipids were adjusted for high-density lipoprotein cholesterol and triglycerides that were significantly associated with genetically predicted sex hormone-binding globulin levels. MVMR-adjusted BPs were adjusted for both systolic and diastolic blood pressures

**Figure 5:**
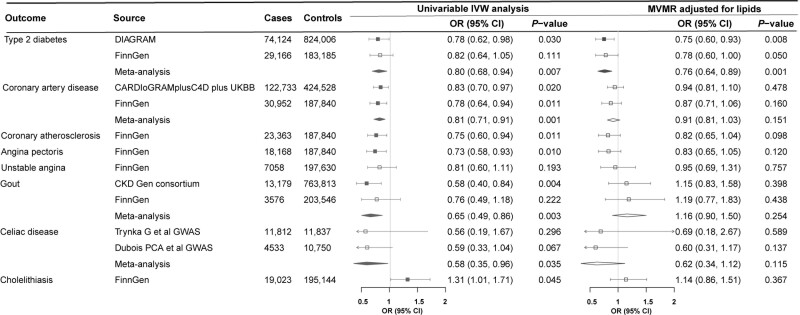
Associations of genetically predicted testosterone levels with diseases in univariable and multivariable Mendelian randomization analysis. CARDIoGRAMplusC4D, Coronary ARtery DIsease Genome wide Replication and Meta-analysis (CARDIoGRAM) plus The Coronary Artery Disease (C4D) Genetics; CI, confidence interval; CKD Gen, Chronic Kidney Disease Genetics; DIAGRAM, DIAbetes Genetics Replication And Meta-analysis; GWAS, genome-wide association study; IVW, inverse variance weighted; MVMR, multivariable Mendelian randomization; OR, odds ratio; UKBB, UK Biobank. MVMR-adjusted lipids were adjusted for low-density and high-density lipoprotein cholesterol and triglycerides that were significantly associated with genetically predicted testosterone levels

Higher genetically predicted SHBG levels were associated with a reduced risk of hypertension, type 2 diabetes, diabetic complications, coronary atherosclerotic outcomes, gout and neoplasm of uterus, and an increased risk of varicose veins and fracture (Figure 4). Higher genetically predicted testosterone levels were associated with a decreased risk of type 2 diabetes, coronary atherosclerotic outcomes, gout and coeliac disease and an increased risk of cholelithiasis (Figure 5). These associations were generally consistent in sensitivity analyses albeit with wider confidence intervals, and moderate to high heterogeneity was observed in most analyses. Horizontal pleiotropy was observed for the associations of genetically predicted SHBG with hypertension and type 2 diabetes, and for the association of genetically predicted testosterone with type 2 diabetes (*P* for MR-Egger intercept <0.05, Supplementary Tables S9 and S10, available as Supplementary data at *IJE* online). No associations were observed for genetically predicted oestradiol levels (Supplementary Table S11, available as Supplementary data at *IJE* online).

### Multivariable MR analysis

Four biomarkers or traits associated with genetically predicted SHBG levels, including high-density lipoprotein cholesterol, triglycerides, blood pressure and uric acid, were adjusted in the multivariable MR analysis for SHBG. Most associations of genetically predicted SHBG, with the exception of type 2 diabetes, gout, fracture and endometrial cancer, were largely attenuated after the adjustment for genetically predicted levels of high-density lipoprotein cholesterol and triglycerides (Figure 4). The improved lipid profile (increased levels of high-density lipoprotein cholesterol and lower levels of triglycerides) mediated most of the protective effects of higher genetically predicted SHBG levels on coronary atherosclerotic outcomes from 78% (95% CI: 30%, 100%) for myocardial infarction to 96% (95% CI: 25%, 100%) for angina pectoris (Supplementary Table S12, available as Supplementary data at *IJE* online). In addition, the associations for hypertension and coronary atherosclerotic outcomes were attenuated in the multivariable MR analyses with adjustment for genetically predicted blood pressure, and the association for gout was attenuated in the multivariable MR analysis with adjustment for genetically predicted uric acid (Figure 4; and Supplementary Tables S12 and S13, available as Supplementary data at *IJE* online). Two biomarkers associated with genetically predicted testosterone levels, including high-density lipoprotein cholesterol and triglycerides, were adjusted in the multivariable MR analysis for testosterone. The associations of genetically predicted testosterone levels with coronary atherosclerotic outcomes and gout were largely attenuated after adjustment for genetically predicted improved lipid profile (Figure 5; and Supplementary Table S14, available as Supplementary data at *IJE* online).

## Discussion

This MR study found that higher genetically predicted SHBG and testosterone levels were associated with the risk of a wide range of health outcomes, and certain associations appeared to be sex specific. An improved blood lipid profile (increased levels of high-density lipoprotein cholesterol and lower levels of triglycerides) appeared to largely mediate the protective effects of higher genetically predicted SHBG and testosterone levels on coronary atherosclerotic diseases.

The inverse association between SHBG and type 2 diabetes is consistent between studies,^41–43^ and females and males,^20^ which supports our findings. Nevertheless, the evidence of effect of SHBG on coronary atherosclerotic outcomes, especially on coronary artery disease, is conflicting.^16^^,^^44^ This study, based on data from UK Biobank, genetic consortia, and FinnGen, revealed consistent inverse associations between higher genetically predicted SHBG levels and a wide range of coronary atherosclerotic diseases in both females and males, which suggests that SHBG may play an important role in atherosclerosis. The inverse association between SHBG and endometrial cancer has been documented in previous studies^18^^,^^20^^,^^45^ and supported by this study. In addition, our analysis added information on the protective effect of high SHBG on benign neoplasm of uterus. The inverse association between SHBG and kidney impairment was observed and in line with a previous MR study,^17^ and we further noticed that high SHBG might prevent or delay kidney stone formations. Another beneficial effect of high SHBG was observed on gout, and this association appeared to be predominant in males (more cases) even though a suggestive inverse association was observed in females as well (not shown). Our two-sample MR study further observed an inverse association between uric acid and genetically predicted SHBG, which supports the novel findings on sex disparity of gout prevalence. Two adverse outcomes, varicose veins and fracture, were identified to be associated with higher levels of genetically predicted SHBG mainly in females. The association for fracture has been indicated in previous observational studies^46^ and the association appears to be mediated by bone mineral density.^47^

The associations of testosterone with potential outcomes have been explored in previous studies. However, the associations are inconsistent, especially for its effects on cardiovascular disease. A review systematically examined observational evidence on the associations of endogenous testosterone with type 2 diabetes, heart failure and coronary artery disease, and found that high testosterone levels were associated with decreased risk of these diseases.^1^ Nevertheless, positive associations of endogenous testosterone with several cardiovascular diseases, including myocardial infarction, heart failure and stroke, were reported in some^19^^,^^48^ but not all MR studies.^14^^,^^16^ Our study observed inverse associations of genetically predicted higher testosterone levels with coronary atherosclerotic diseases mainly in males, and confirmed these associations in two-sample MR analyses without sex-specific data. Evidence from randomized clinical trials concluded differently on the association between testosterone replacement therapy and cardiovascular risk,^9^^,^^10^ and a recent review suggested that no published trials of testosterone replacement therapy were adequately powered to assess cardiovascular events.^49^ We additionally observed a protective effect of higher genetically predicted testosterone levels with type 2 diabetes, a consistent finding with previous MR studies.^20^^,^^41^ Higher testosterone appeared to lower risk of gout and coeliac disease mainly in males and to increase risk of gallstones in females, which are novel findings that need verification. A previous two-sample MR analysis showed a positive association between genetically predicted testosterone levels and risk of prostate cancer^20^ which was not confirmed in the present MR study, possibly because of lack of power.

Endogenous SHBG and testosterone have been associated with metabolic features,^50^^,^^51^ although evidence on effects of testosterone replacement therapy is inconsistent between females and males.^9^^,^^52^ Our findings on the associations of genetically predicted SHBG with lipid metabolism are in line with a previous phenome-wide MR on SHBG proxied by 10 SNPs.^15^ However, the observed inverse association of genetically predicted testosterone with high-density lipoprotein cholesterol in males was not in agreement with another MR study.^14^ Except for lipids, our MR analyses hinted that SHBG might exert protective effects on other metabolic factors, such as blood pressure as well as urate levels. Our multivariable MR analysis observed attenuated associations mainly for coronary atherosclerotic diseases after adjustment for genetically predicted high-density lipoprotein cholesterol and triglycerides. These findings suggest that the improved blood lipid profile featured by higher levels of high-density lipoprotein cholesterol and lower levels of triglycerides mediated the observed associations between SHBG and testosterone and cardiovascular diseases. Findings from mediation analyses may provide hints for underlying mechanisms behind the protective effects of high endogenous SHBG and testosterone levels.

Oestradiol has been inconsistently associated with cardiovascular risk in females and males in previous observational studies.^44^^,^^53^ Oestrogen therapy showed associations with diabetes, stroke, venous thromboembolism, fracture and cancer in randomized clinical trials in females.^11^ Given that genetic instruments for oestradiol were selected in males (oestradiol levels were too low to be detected in the majority of studied females),^20^ we did not perform analysis for oestradiol in females because genetic instruments used for oestradiol were not associated with oestradiol levels in premenopausal females in UK Biobank. However, we observed associations of higher genetically predicted oestradiol levels with lower risk of peripheral vascular disease and polyarteritis nodosa in males. Even though we did not replicate these associations in two-sample MR analysis, we could not completely rule out the causality of these associations in males only, since no sex-specific analysis was conducted in two-sample MR analysis.

The major strength of the present study is that we examined the associations between SHBG, testosterone and oestradiol and a broad spectrum of disease outcomes, using updated instruments in UK Biobank. In addition, we conducted phenome-wide MR analyses stratified by sex. Most associations were replicated using two-sample MR analyses with outcome data from different genetic studies and consortia,^22–31^ and the consistency of results strengthened the observed associations. In addition, multivariable MR analyses were performed to detect the mediations of hormone-associated biomarkers on observed associations for genetically predicted SHBG and testosterone. Mediation analysis not only supported the associations of SHBG and testosterone with disease endpoints, but also implied possible biological mechanisms. Last, we confined most studied populations to individuals of European ancestry, which minimized the population structure bias. However, this population confinement limited the generalizability of our findings to other populations.

This study has several limitations that need consideration when interpreting our results. Pleiotropy is a major concern given that we used multiple genetic instruments for exposures. Even though we performed several sensitivity analyses and multivariable MR analyses, and detected minimal horizontal pleiotropy in most associations, we could not completely rule out the possibility that unobserved pleiotropic effects might bias the established associations or conceal the relatively weak causal effects of SHBG and sex hormones on diseases. In addition, instrumental variables were extracted from a genome-wide association study in UK Biobank, the outcome data source of phenome-wide MR analysis, which might bias the causal estimates towards observational associations.^54^ However, most associations were successfully replicated in two-sample MR analyses. Besides, we examined the strength of instruments used in UK Biobank, and the F statistics were >10. In phenome-wide MR analysis, most cases were identified from the inpatient hospital records, which might compromise the coverage of case ascertainment, especially for the diseases that do not usually need hospitalization. Although the incorporation of self-reported data would improve this limitation, it is likely to include patients without a true diagnosis and therefore to introduce information bias. With balanced consideration between the study power and the number of health outcomes to be analysed, we used the variance explained by SHBG instruments which was larger than that of testosterone and oestradiol in power estimation to determine the minimum number of cases. It should be noted that this would increase the false negative probability of phenome-wide MR findings for testosterone or oestradiol, as the analyses might not have enough power to detect disease outcomes with the small number of cases. We used genetic instruments to mimic endogenous levels of SHBG and sex hormones in females and males separately in the phenome-wide MR analysis; however, given that no sex-specific data were available in the replication datasets, sex-specific two-sample MR analysis could not be conducted. In addition, our findings may not accurately predict, but may provide clues to, the potential effects of hormone replacement therapy.

## Conclusion

This MR study suggests robust associations of SHBG with a wide range of diseases, including hypertension, type 2 diabetes, diabetic complications, coronary atherosclerotic outcomes, gout, benign and malignant neoplasm of uterus, varicose veins and fracture. Higher testosterone levels appear to lower risk of type 2 diabetes, coronary atherosclerotic outcomes, gout and coeliac disease in males, and to increase risk of cholelithiasis in females. These findings support that SHBG may act as a causal factor associated with above diseases, and they strengthen the case for strategies that improve endogenous SHBG and testosterone levels for disease prevention.

## Ethics approval

UK Biobank received ethical permits from the North West Multi-centre Research Ethics Committee, the National Information Governance Board for Health and Social Care in England and Wales and the Community Health Index Advisory Group in Scotland. All participants provided written informed consent.

## Supplementary Material

dyac036_Supplementary_Data

## Data Availability

Data used in this study can be obtained by a reasonable request to corresponding author. This work has been conducted using the UK Biobank Resource. The UK Biobank is an open access resource and bona fide researchers can apply to use the UK Biobank dataset by registering and applying at [http://ukbiobank.ac.uk/register-apply/].
