# Supplementary material for: Genetically predicted sex hormone levels and health outcomes: phenome-wide Mendelian randomization investigation
Source: Int J Epidemiol. Author manuscript; Available in PMC 2022 Dec 15. (PMC9749729; doi:10.1093/ije/dyac036)
Supplement: Supplementary File [file EMS157110-supplement-Supplementary_File.docx]

Supporting information for

**Genetically Predicted Sex Hormone Levels and Health Outcomes: Phenome-wide Mendelian Randomization Investigation**

*Shuai Yuan, Lijuan Wang, Jing Sun, Lili Yu, Xuan Zhou, Jie Yang, Yimin Zhu, Dipender Gill, Stephen Burgess,* *Joshua C Denny, Susanna C. Larsson, Evropi Theodoratou, Xue Li*

**Supplementary method**

**Supplementary Table 1.** Used genetic instruments

**Supplementary Table 2.** Information on genome-wide analysis on sex hormones in the UK Biobank study

**Supplementary Table 3**. Information on used consortia and studies in Mendelian randomization analysis

**Supplementary Table 4.** Outcomes included in the analyses and outcomes excluded due to power (N<120)

**Supplementary Table 4.** Characteristics of participants in the UK Biobank

**Supplementary Table 5.** Outcomes included in the analyses and outcomes excluded due to power (N<120)

**Supplementary Table 6.** Disease outcomes associated with the weighted genetic risk score of sex hormone binding globulin in phenome-wide MR analysis

**Supplementary Table 7.** Disease outcomes associated with the weighted genetic risk score of testosterone in phenome-wide MR analysis

**Supplementary Table 8.** Disease outcomes associated with the weighted genetic risk score of estradiol in phenome-wide MR analysis

**Supplementary Table 9.** Associations of genetically predicted sex hormone-binding globulin levels with diseases and biomarkers in Mendelian randomization sensitivity analyses

**Supplementary Table 10.** Associations of genetically predicted testosterone levels with diseases and biomarkers in Mendelian randomization sensitivity analyses

**Supplementary Table 11.** Associations of genetically predicted estradiol levels with diseases and biomarkers in Mendelian randomization analyses

**Supplementary Table 12.** Mediation effects in two-sample MR analyses for genetically predicted sex hormone-binding globulin levels

**Supplementary Table 13.** Associations of genetically predicted sex hormone-binding globulin levels with diseases in univariable and multivariable Mendelian randomization analysis

**Supplementary Table 14.** Mediation effects in two-sample MR analyses for genetically predicted testosterone levels

**Supplementary Figure 1.** Power calculations for the clinical endpoints (OR>2 or OR <0.5 at the significance level of α=0.05 and assuming phenotypic variance at 6.9% (corresponding to the variance explained by genetic variants associated with SHBG levels)).

**Supplementary Figure 2**. Associations of genetically predicted estradiol levels with clinical outcomes and biomarkers in the phenome-wide association analysis in the UK Biobank.

**Supplementary method**

**UK Biobank data**

***Genotype data*** - Genotyping, quality control and genotype imputation were conducted by the UK Biobank team prior to the data release and the exact procedure is described by Bycroft *et al* [1]*.* The initial 50 000 participants were genotyped by the Affymetrix UK BiLEVE Axiom array and the remaining 45 000 participants were genotyped by the Affymetrix UK Biobank Axiom array. Genotype imputation was performed based on a merged reference panel of the Haplotype Reference Consortium (HRC) [2] and the UK10K haplotype resources [3], and the classical allelic variations at the MHC region were further imputed by using an additional multi-population reference panel [4]. For quality control, a list of field variables was made available by the UK Biobank to indicate the genotype quality, population structure, and genetic relatedness.

***Phenotype data*** - A variety of national health systems and sources were used by the UK Biobank to follow up the disease diagnosis, cancer occurrence, and causes of death among the enrolled participants. Currently, there are three main different types of health records (i.e., hospital inpatient episodes, cancer registry data and death registry data) that have been incorporated into the central database. The coding for clinical diagnoses in these datasets followed the World Health Organization’s International Classification of Diseases (ICD) coding systems but used different ICD versions (ICD-10 or ICD-9) according to the date of the record. Primary and/or secondary ICD codes are available in the hospital inpatient data and/or death registry data to classify the main causes and contributory causes of the event of hospitalization and/or death respectively.

**Study population and quality control**

To minimize the influence of the diverse population structure in UK Biobank, our study was constrained to a subset of unrelated White British subjects with high quality genotype data. The metrics used for genotype quality control (QC) were based on the data fields created by the UK Biobank. Samples that were identified as a sex mismatch, outliers with high heterozygosity or with high missing rate, putative aneuploidy in sex chromosome, individuals with excess relatives, or non-White British ancestry were all excluded from the analysis. The largest possible subset of individuals without relatedness were identified using an algorithm implemented in the R package “*i-graph (v1.0.1)*” developed by Bycroft and colleagues [1].

**Reference**

1. Bycroft C, Freeman C, Petkova D, Band G, Elliott LT, Sharp K, et al. Genome-wide genetic data on~ 500,000 UK Biobank participants. bioRxiv. 2017.
2. McCarthy S, Das S, Kretzschmar W, Delaneau O, Wood AR, Teumer A, et al. A reference panel of 64,976 haplotypes for genotype imputation. Nat Genet. 2016;48(10):1279-83.
3. Walter K, Min JL, Huang J, Crooks L, Memari Y, McCarthy S, et al. The UK10K project identifies rare variants in health and disease. Nature. 2015;526(7571):82-90.
4. Dilthey A, Leslie S, Moutsianas L, Shen J, Cox C, Nelson MR, et al. Multi-population classical HLA type imputation. PLoS Comput Biol. 2013;9(2):e1002877.

**Supplementary Table 1.** Used genetic instruments

| **Hormone** | **SNP** | **Chromosome** | **Position** | **Effect allele** | **Other allele** | **EAF** | **Beta** | **SE** | **P val** |
| --- | --- | --- | --- | --- | --- | --- | --- | --- | --- |
| Estradiol | rs1260326 | 2 | 27730940 | C | T | 0.606 | 0.006 | 0.001 | 9.60E-11 |
| Estradiol | rs112881196 | 2 | 31982811 | G | C | 0.039 | 0.025 | 0.002 | 5.60E-30 |
| Estradiol | rs45446698 | 7 | 99332948 | T | G | 0.958 | 0.016 | 0.002 | 7.90E-14 |
| Estradiol | rs657152 | 9 | 136139265 | C | A | 0.661 | 0.008 | 0.001 | 5.50E-21 |
| Estradiol | rs56196860 | 12 | 2908330 | A | C | 0.031 | 0.021 | 0.002 | 2.60E-18 |
| Estradiol | rs34019140 | 14 | 106527500 | G | A | 0.564 | 0.012 | 0.001 | 6.90E-42 |
| Estradiol | rs7173595 | 15 | 51533736 | T | C | 0.649 | 0.016 | 0.001 | 3.60E-72 |
| Estradiol | rs3751591 | 15 | 51606710 | G | A | 0.167 | 0.008 | 0.001 | 6.60E-12 |
| Estradiol | rs727428 | 17 | 7537792 | C | T | 0.557 | 0.006 | 0.001 | 1.80E-11 |
| Estradiol | rs113047993 | 18 | 20585399 | C | T | 0.931 | 0.010 | 0.002 | 8.70E-09 |
| Estradiol | rs201687269 | 18 | 27520799 | T | A | 0.675 | 0.006 | 0.001 | 1.80E-08 |
| Estradiol | rs10425629 | 19 | 48384648 | C | T | 0.165 | 0.008 | 0.001 | 2.70E-11 |
| Estradiol | rs117826558 | 22 | 46770756 | T | C | 0.034 | 0.014 | 0.002 | 2.70E-08 |
| SHBG | rs7539725 | 1 | 10125407 | G | A | 0.137 | 0.007 | 0.001 | 8.00E-10 |
| SHBG | rs75077113 | 1 | 11214582 | C | A | 0.277 | 0.009 | 0.001 | 2.40E-22 |
| SHBG | rs198358 | 1 | 11904076 | C | T | 0.248 | 0.007 | 0.001 | 6.90E-13 |
| SHBG | rs36086195 | 1 | 16510894 | T | C | 0.580 | 0.011 | 0.001 | 2.90E-43 |
| SHBG | rs114165349 | 1 | 27021913 | G | C | 0.977 | 0.081 | 0.003 | 1.00E-200 |
| SHBG | rs111642750 | 1 | 29320013 | A | G | 0.051 | 0.011 | 0.002 | 8.10E-10 |
| SHBG | rs947643 | 1 | 31461438 | G | A | 0.752 | 0.005 | 0.001 | 2.30E-08 |
| SHBG | rs1969213 | 1 | 42238531 | A | G | 0.528 | 0.005 | 0.001 | 3.40E-11 |
| SHBG | rs2782640 | 1 | 44009033 | T | C | 0.622 | 0.005 | 0.001 | 3.50E-09 |
| SHBG | rs1883783 | 1 | 54890956 | T | G | 0.430 | 0.006 | 0.001 | 6.50E-14 |
| SHBG | rs35067979 | 1 | 61925076 | C | T | 0.929 | 0.015 | 0.002 | 2.20E-17 |
| SHBG | rs469864 | 1 | 91542517 | T | C | 0.791 | 0.009 | 0.001 | 6.40E-17 |
| SHBG | rs12385720 | 1 | 93539383 | G | A | 0.371 | 0.007 | 0.001 | 6.60E-19 |
| SHBG | rs1730859 | 1 | 107617707 | G | A | 0.343 | 0.026 | 0.001 | 6.60E-197 |
| SHBG | rs140584594 | 1 | 110232983 | A | G | 0.270 | 0.013 | 0.001 | 3.60E-49 |
| SHBG | rs41264630 | 1 | 149885800 | A | G | 0.080 | 0.013 | 0.002 | 7.20E-20 |
| SHBG | rs267733 | 1 | 150958836 | A | G | 0.839 | 0.012 | 0.001 | 6.60E-29 |
| SHBG | rs72694845 | 1 | 151909812 | A | C | 0.970 | 0.017 | 0.002 | 2.10E-12 |
| SHBG | rs9427104 | 1 | 154589232 | T | C | 0.479 | 0.012 | 0.001 | 8.60E-53 |
| SHBG | rs10797877 | 1 | 171075556 | C | T | 0.529 | 0.006 | 0.001 | 1.20E-15 |
| SHBG | rs12138803 | 1 | 172348823 | C | T | 0.730 | 0.006 | 0.001 | 2.30E-10 |
| SHBG | rs2274432 | 1 | 184020945 | G | A | 0.653 | 0.005 | 0.001 | 2.50E-11 |
| SHBG | rs78444298 | 1 | 184672098 | G | A | 0.980 | 0.025 | 0.003 | 1.80E-18 |
| SHBG | rs4639796 | 1 | 197126649 | G | A | 0.840 | 0.012 | 0.001 | 4.30E-28 |
| SHBG | rs17583875 | 1 | 197924770 | A | G | 0.021 | 0.027 | 0.003 | 2.70E-23 |
| SHBG | rs7540115 | 1 | 200265618 | C | A | 0.819 | 0.007 | 0.001 | 6.00E-12 |
| SHBG | rs2802770 | 1 | 203518456 | A | T | 0.537 | 0.007 | 0.001 | 5.90E-15 |
| SHBG | rs2369633 | 1 | 205181062 | T | C | 0.092 | 0.011 | 0.001 | 3.50E-15 |
| SHBG | rs1418652 | 1 | 205646458 | C | T | 0.386 | 0.006 | 0.001 | 6.50E-12 |
| SHBG | rs2456827 | 1 | 212942595 | T | C | 0.786 | 0.005 | 0.001 | 2.00E-08 |
| SHBG | rs1223791 | 1 | 214321081 | G | A | 0.163 | 0.015 | 0.001 | 3.30E-44 |
| SHBG | rs7539006 | 1 | 214398058 | A | C | 0.255 | 0.006 | 0.001 | 1.60E-11 |
| SHBG | rs3001032 | 1 | 219727779 | C | T | 0.320 | 0.011 | 0.001 | 1.30E-37 |
| SHBG | rs2247213 | 1 | 221055463 | G | A | 0.670 | 0.012 | 0.001 | 1.10E-45 |
| SHBG | rs2234922 | 1 | 226026406 | A | G | 0.800 | 0.008 | 0.001 | 6.80E-13 |
| SHBG | rs1870927 | 1 | 226426337 | A | T | 0.621 | 0.007 | 0.001 | 3.70E-18 |
| SHBG | rs3887753 | 1 | 227695517 | C | T | 0.177 | 0.007 | 0.001 | 2.60E-11 |
| SHBG | rs13402475 | 2 | 3639909 | C | G | 0.185 | 0.006 | 0.001 | 3.00E-08 |
| SHBG | rs4668732 | 2 | 11716919 | T | A | 0.637 | 0.006 | 0.001 | 2.90E-13 |
| SHBG | rs11096542 | 2 | 18707873 | G | A | 0.407 | 0.005 | 0.001 | 5.40E-11 |
| SHBG | rs35633876 | 2 | 20363074 | G | T | 0.516 | 0.008 | 0.001 | 2.90E-23 |
| SHBG | rs62130499 | 2 | 27184873 | A | C | 0.088 | 0.010 | 0.001 | 6.70E-12 |
| SHBG | rs4665972 | 2 | 27598097 | C | T | 0.607 | 0.037 | 0.001 | 1.00E-200 |
| SHBG | rs10210970 | 2 | 28646847 | C | T | 0.871 | 0.009 | 0.001 | 7.90E-15 |
| SHBG | rs72798731 | 2 | 32515337 | T | C | 0.034 | 0.014 | 0.002 | 7.20E-09 |
| SHBG | rs56219475 | 2 | 39241107 | A | G | 0.009 | 0.026 | 0.005 | 1.00E-09 |
| SHBG | rs6736913 | 2 | 42510018 | A | G | 0.021 | 0.032 | 0.003 | 2.30E-32 |
| SHBG | rs11690748 | 2 | 48584575 | C | G | 0.623 | 0.006 | 0.001 | 7.30E-15 |
| SHBG | rs998230 | 2 | 55121930 | C | G | 0.608 | 0.005 | 0.001 | 2.90E-09 |
| SHBG | rs17008851 | 2 | 61606097 | G | A | 0.133 | 0.010 | 0.001 | 2.10E-16 |
| SHBG | rs6546096 | 2 | 64906295 | A | G | 0.262 | 0.021 | 0.001 | 3.00E-110 |
| SHBG | rs11902527 | 2 | 66210213 | A | G | 0.676 | 0.005 | 0.001 | 2.00E-08 |
| SHBG | rs12624244 | 2 | 70417138 | A | G | 0.936 | 0.020 | 0.002 | 7.60E-33 |
| SHBG | rs2670747 | 2 | 71525784 | G | A | 0.127 | 0.007 | 0.001 | 2.50E-10 |
| SHBG | rs11164095 | 2 | 97155208 | T | C | 0.279 | 0.006 | 0.001 | 4.10E-10 |
| SHBG | rs3747647 | 2 | 112245586 | C | G | 0.218 | 0.009 | 0.001 | 3.70E-19 |
| SHBG | rs3979376 | 2 | 113225155 | A | G | 0.475 | 0.004 | 0.001 | 1.30E-09 |
| SHBG | rs10211038 | 2 | 114593805 | A | G | 0.189 | 0.007 | 0.001 | 2.30E-10 |
| SHBG | rs11688682 | 2 | 121347612 | C | G | 0.271 | 0.007 | 0.001 | 1.90E-16 |
| SHBG | rs2307394 | 2 | 148716428 | C | T | 0.303 | 0.006 | 0.001 | 7.10E-12 |
| SHBG | rs13389219 | 2 | 165528876 | T | C | 0.393 | 0.016 | 0.001 | 1.60E-88 |
| SHBG | rs10187560 | 2 | 173925051 | T | C | 0.303 | 0.005 | 0.001 | 8.90E-10 |
| SHBG | rs2364717 | 2 | 178101235 | T | C | 0.539 | 0.006 | 0.001 | 3.30E-16 |
| SHBG | rs10169561 | 2 | 180458771 | C | T | 0.464 | 0.005 | 0.001 | 6.30E-09 |
| SHBG | rs1047891 | 2 | 211540507 | A | C | 0.316 | 0.010 | 0.001 | 1.20E-29 |
| SHBG | rs2014998 | 2 | 217681961 | A | G | 0.260 | 0.005 | 0.001 | 3.20E-08 |
| SHBG | rs62182125 | 2 | 219274142 | G | A | 0.449 | 0.009 | 0.001 | 1.50E-25 |
| SHBG | rs78058190 | 2 | 219699999 | G | A | 0.950 | 0.021 | 0.002 | 2.00E-24 |
| SHBG | rs57467915 | 2 | 220081416 | G | A | 0.985 | 0.022 | 0.003 | 3.90E-11 |
| SHBG | rs4674669 | 2 | 223430985 | C | T | 0.139 | 0.007 | 0.001 | 9.10E-09 |
| SHBG | rs2943641 | 2 | 227093745 | T | C | 0.353 | 0.014 | 0.001 | 9.80E-66 |
| SHBG | rs11682084 | 2 | 231286503 | C | A | 0.714 | 0.006 | 0.001 | 3.20E-13 |
| SHBG | rs62195072 | 2 | 234260879 | C | T | 0.324 | 0.008 | 0.001 | 3.30E-18 |
| SHBG | rs28898590 | 2 | 234619422 | G | T | 0.934 | 0.011 | 0.002 | 5.20E-11 |
| SHBG | rs62193162 | 2 | 242268436 | T | A | 0.639 | 0.005 | 0.001 | 3.30E-09 |
| SHBG | rs1801282 | 3 | 12393125 | G | C | 0.120 | 0.022 | 0.001 | 3.00E-67 |
| SHBG | rs6792725 | 3 | 24520283 | G | A | 0.693 | 0.015 | 0.001 | 1.10E-63 |
| SHBG | rs784504 | 3 | 39195260 | C | G | 0.811 | 0.007 | 0.001 | 4.00E-14 |
| SHBG | rs12487736 | 3 | 47459679 | C | T | 0.424 | 0.009 | 0.001 | 3.90E-27 |
| SHBG | rs1965132 | 3 | 69147519 | C | A | 0.505 | 0.005 | 0.001 | 1.90E-10 |
| SHBG | rs2597305 | 3 | 70922562 | G | C | 0.454 | 0.005 | 0.001 | 7.30E-10 |
| SHBG | rs13082048 | 3 | 86964322 | A | G | 0.361 | 0.006 | 0.001 | 2.60E-10 |
| SHBG | rs958650 | 3 | 98736153 | G | A | 0.770 | 0.006 | 0.001 | 6.90E-09 |
| SHBG | rs13315174 | 3 | 105406468 | G | A | 0.784 | 0.008 | 0.001 | 6.00E-17 |
| SHBG | rs6776396 | 3 | 124321704 | T | C | 0.246 | 0.005 | 0.001 | 4.50E-08 |
| SHBG | rs2953761 | 3 | 129745975 | A | G | 0.776 | 0.006 | 0.001 | 3.30E-10 |
| SHBG | rs62292950 | 3 | 132197995 | T | G | 0.868 | 0.006 | 0.001 | 1.80E-08 |
| SHBG | rs687339 | 3 | 135932359 | C | T | 0.228 | 0.027 | 0.001 | 1.30E-175 |
| SHBG | rs6777420 | 3 | 142002869 | T | C | 0.131 | 0.007 | 0.001 | 8.80E-09 |
| SHBG | rs9834503 | 3 | 149994882 | A | C | 0.539 | 0.006 | 0.001 | 1.70E-11 |
| SHBG | rs62271373 | 3 | 150066540 | T | A | 0.940 | 0.018 | 0.002 | 2.30E-24 |
| SHBG | rs55735727 | 3 | 169488148 | T | A | 0.269 | 0.007 | 0.001 | 2.60E-13 |
| SHBG | rs10936702 | 3 | 171548897 | A | G | 0.394 | 0.005 | 0.001 | 7.90E-09 |
| SHBG | rs79287178 | 3 | 172294500 | G | A | 0.969 | 0.037 | 0.003 | 1.70E-51 |
| SHBG | rs234043 | 3 | 172313367 | T | C | 0.282 | 0.009 | 0.001 | 1.10E-27 |
| SHBG | rs2293606 | 3 | 184106493 | G | T | 0.056 | 0.011 | 0.002 | 7.50E-10 |
| SHBG | rs57158761 | 3 | 185371172 | A | G | 0.564 | 0.009 | 0.001 | 1.70E-27 |
| SHBG | rs7430950 | 3 | 196233136 | A | C | 0.747 | 0.006 | 0.001 | 3.60E-11 |
| SHBG | rs11725653 | 4 | 963397 | C | T | 0.894 | 0.011 | 0.001 | 2.90E-17 |
| SHBG | rs13108218 | 4 | 3443931 | A | G | 0.383 | 0.024 | 0.001 | 3.00E-172 |
| SHBG | rs4450871 | 4 | 4990298 | G | A | 0.442 | 0.006 | 0.001 | 6.40E-14 |
| SHBG | rs2724475 | 4 | 17946432 | T | C | 0.262 | 0.007 | 0.001 | 5.20E-14 |
| SHBG | rs2970877 | 4 | 23887454 | G | T | 0.297 | 0.009 | 0.001 | 1.50E-20 |
| SHBG | rs28473232 | 4 | 37406477 | G | A | 0.232 | 0.006 | 0.001 | 2.60E-09 |
| SHBG | rs2381145 | 4 | 38452427 | C | T | 0.799 | 0.006 | 0.001 | 2.70E-08 |
| SHBG | rs6531735 | 4 | 39686332 | G | A | 0.493 | 0.005 | 0.001 | 1.00E-09 |
| SHBG | rs7696472 | 4 | 69538180 | A | G | 0.475 | 0.013 | 0.001 | 8.10E-58 |
| SHBG | rs1881668 | 4 | 70725456 | G | C | 0.269 | 0.005 | 0.001 | 1.10E-08 |
| SHBG | rs28507491 | 4 | 77197651 | A | G | 0.377 | 0.013 | 0.001 | 1.20E-51 |
| SHBG | rs116243488 | 4 | 83245180 | T | G | 0.085 | 0.008 | 0.002 | 3.90E-08 |
| SHBG | rs13150068 | 4 | 88203828 | A | G | 0.563 | 0.018 | 0.001 | 1.40E-112 |
| SHBG | rs7678138 | 4 | 120106766 | G | A | 0.874 | 0.011 | 0.001 | 1.70E-18 |
| SHBG | rs1433210 | 4 | 124766956 | C | A | 0.246 | 0.008 | 0.001 | 2.90E-17 |
| SHBG | rs62334584 | 4 | 129031788 | T | C | 0.621 | 0.007 | 0.001 | 6.10E-16 |
| SHBG | rs28925904 | 4 | 144359490 | C | T | 0.975 | 0.021 | 0.003 | 3.60E-16 |
| SHBG | rs75686861 | 4 | 145621328 | G | A | 0.908 | 0.008 | 0.001 | 3.80E-10 |
| SHBG | rs10027275 | 4 | 148981496 | G | C | 0.259 | 0.011 | 0.001 | 5.70E-36 |
| SHBG | rs28367132 | 4 | 149639865 | A | G | 0.203 | 0.007 | 0.001 | 4.80E-11 |
| SHBG | rs72729610 | 4 | 154190965 | A | G | 0.833 | 0.010 | 0.001 | 8.10E-18 |
| SHBG | rs28730491 | 4 | 157681274 | C | G | 0.319 | 0.009 | 0.001 | 3.40E-24 |
| SHBG | rs78890745 | 4 | 159834474 | A | G | 0.109 | 0.017 | 0.001 | 1.70E-37 |
| SHBG | rs11729169 | 4 | 171019391 | T | C | 0.107 | 0.011 | 0.001 | 2.80E-15 |
| SHBG | rs11721999 | 4 | 185377222 | G | C | 0.165 | 0.007 | 0.001 | 9.90E-11 |
| SHBG | rs72709458 | 5 | 1283755 | C | T | 0.793 | 0.006 | 0.001 | 1.50E-09 |
| SHBG | rs4266430 | 5 | 38749736 | T | C | 0.488 | 0.005 | 0.001 | 1.50E-09 |
| SHBG | rs7735249 | 5 | 53310139 | C | G | 0.887 | 0.019 | 0.001 | 3.90E-46 |
| SHBG | rs40270 | 5 | 55804552 | A | C | 0.227 | 0.016 | 0.001 | 2.30E-60 |
| SHBG | rs79354983 | 5 | 56221537 | A | G | 0.905 | 0.012 | 0.001 | 5.20E-19 |
| SHBG | rs72753349 | 5 | 57392079 | T | C | 0.041 | 0.011 | 0.002 | 2.90E-08 |
| SHBG | rs4976033 | 5 | 67714246 | A | G | 0.599 | 0.007 | 0.001 | 9.70E-20 |
| SHBG | rs34651 | 5 | 72144005 | T | C | 0.919 | 0.012 | 0.002 | 8.60E-16 |
| SHBG | rs335629 | 5 | 76729521 | A | G | 0.485 | 0.004 | 0.001 | 1.10E-08 |
| SHBG | rs58729412 | 5 | 90169070 | C | T | 0.281 | 0.007 | 0.001 | 1.70E-13 |
| SHBG | rs13165542 | 5 | 122769296 | T | C | 0.824 | 0.008 | 0.001 | 6.70E-13 |
| SHBG | rs6860245 | 5 | 127367998 | C | G | 0.248 | 0.011 | 0.001 | 3.20E-30 |
| SHBG | rs2057655 | 5 | 131807624 | A | G | 0.186 | 0.010 | 0.001 | 2.80E-20 |
| SHBG | rs329120 | 5 | 133861756 | C | T | 0.581 | 0.009 | 0.001 | 2.30E-27 |
| SHBG | rs72802806 | 5 | 142798588 | G | A | 0.770 | 0.006 | 0.001 | 8.60E-09 |
| SHBG | rs2431752 | 5 | 162882702 | A | G | 0.107 | 0.010 | 0.001 | 3.50E-14 |
| SHBG | rs75049939 | 5 | 173334219 | T | G | 0.695 | 0.007 | 0.001 | 7.20E-18 |
| SHBG | rs1128287 | 5 | 176728458 | T | G | 0.777 | 0.005 | 0.001 | 1.80E-09 |
| SHBG | rs9379084 | 6 | 7231843 | G | A | 0.884 | 0.014 | 0.001 | 5.20E-26 |
| SHBG | rs2299055 | 6 | 15398331 | A | G | 0.118 | 0.007 | 0.001 | 1.00E-08 |
| SHBG | rs10946313 | 6 | 19381386 | T | C | 0.630 | 0.005 | 0.001 | 1.80E-11 |
| SHBG | rs4052755 | 6 | 19914496 | T | C | 0.292 | 0.006 | 0.001 | 1.40E-11 |
| SHBG | rs1408270 | 6 | 25873184 | G | A | 0.268 | 0.007 | 0.001 | 5.10E-16 |
| SHBG | rs9266184 | 6 | 31324664 | T | G | 0.681 | 0.011 | 0.001 | 3.30E-38 |
| SHBG | rs9461976 | 6 | 34237199 | G | A | 0.970 | 0.017 | 0.003 | 6.30E-10 |
| SHBG | rs4135240 | 6 | 36647680 | C | T | 0.330 | 0.007 | 0.001 | 3.00E-15 |
| SHBG | rs28360642 | 6 | 41667506 | A | C | 0.840 | 0.016 | 0.001 | 8.70E-48 |
| SHBG | rs1570360 | 6 | 43737830 | A | G | 0.329 | 0.006 | 0.001 | 2.70E-10 |
| SHBG | rs998584 | 6 | 43757896 | C | A | 0.518 | 0.009 | 0.001 | 2.10E-31 |
| SHBG | rs62407923 | 6 | 52391951 | T | C | 0.172 | 0.007 | 0.001 | 3.40E-09 |
| SHBG | rs12662365 | 6 | 80905389 | A | T | 0.321 | 0.005 | 0.001 | 8.00E-09 |
| SHBG | rs4472353 | 6 | 96078826 | C | T | 0.842 | 0.007 | 0.001 | 1.20E-08 |
| SHBG | rs17185536 | 6 | 100620931 | T | C | 0.244 | 0.007 | 0.001 | 1.70E-14 |
| SHBG | rs11153046 | 6 | 107441753 | A | G | 0.323 | 0.005 | 0.001 | 7.40E-10 |
| SHBG | rs9480889 | 6 | 109189021 | C | G | 0.217 | 0.006 | 0.001 | 2.10E-08 |
| SHBG | rs150115323 | 6 | 117506408 | G | C | 0.372 | 0.005 | 0.001 | 8.50E-12 |
| SHBG | rs6916491 | 6 | 119247734 | T | C | 0.707 | 0.005 | 0.001 | 2.10E-08 |
| SHBG | rs58321169 | 6 | 126868567 | C | T | 0.733 | 0.010 | 0.001 | 1.60E-25 |
| SHBG | rs12661232 | 6 | 130379160 | T | C | 0.312 | 0.011 | 0.001 | 7.70E-34 |
| SHBG | rs2908522 | 6 | 139835399 | G | C | 0.585 | 0.006 | 0.001 | 6.50E-12 |
| SHBG | rs11155787 | 6 | 151686905 | C | T | 0.360 | 0.006 | 0.001 | 1.10E-12 |
| SHBG | rs668871 | 6 | 160769811 | T | C | 0.469 | 0.018 | 0.001 | 1.40E-111 |
| SHBG | rs6919154 | 6 | 163743975 | A | G | 0.591 | 0.006 | 0.001 | 6.70E-12 |
| SHBG | rs4709746 | 6 | 164133001 | T | C | 0.134 | 0.010 | 0.001 | 6.40E-17 |
| SHBG | rs71538127 | 7 | 1010801 | C | G | 0.877 | 0.009 | 0.001 | 1.50E-13 |
| SHBG | rs62442919 | 7 | 1978384 | A | G | 0.382 | 0.008 | 0.001 | 1.50E-23 |
| SHBG | rs2462661 | 7 | 6702311 | G | T | 0.575 | 0.007 | 0.001 | 1.00E-16 |
| SHBG | rs38197 | 7 | 15909008 | C | G | 0.734 | 0.007 | 0.001 | 4.50E-13 |
| SHBG | rs2723572 | 7 | 17888587 | T | C | 0.488 | 0.005 | 0.001 | 3.20E-11 |
| SHBG | rs10951130 | 7 | 26385826 | T | G | 0.855 | 0.008 | 0.001 | 1.80E-12 |
| SHBG | rs10225221 | 7 | 35401635 | C | A | 0.299 | 0.005 | 0.001 | 3.10E-08 |
| SHBG | rs1799831 | 7 | 44199142 | C | T | 0.844 | 0.009 | 0.001 | 1.60E-13 |
| SHBG | rs7794048 | 7 | 46275882 | G | A | 0.937 | 0.013 | 0.002 | 4.80E-14 |
| SHBG | rs6975610 | 7 | 46642734 | G | A | 0.448 | 0.006 | 0.001 | 4.50E-11 |
| SHBG | rs12536766 | 7 | 70158864 | T | G | 0.570 | 0.006 | 0.001 | 4.10E-13 |
| SHBG | rs11770446 | 7 | 77358072 | G | A | 0.288 | 0.007 | 0.001 | 1.40E-19 |
| SHBG | rs1229492 | 7 | 81564122 | T | C | 0.268 | 0.011 | 0.001 | 1.30E-32 |
| SHBG | rs445 | 7 | 92408370 | C | T | 0.905 | 0.012 | 0.001 | 4.80E-18 |
| SHBG | rs7015 | 7 | 97920623 | G | A | 0.815 | 0.034 | 0.001 | 1.00E-200 |
| SHBG | rs1859690 | 7 | 99227172 | G | A | 0.067 | 0.013 | 0.002 | 7.10E-16 |
| SHBG | rs12667888 | 7 | 100474289 | C | T | 0.183 | 0.017 | 0.001 | 2.10E-59 |
| SHBG | rs149092986 | 7 | 111624089 | T | C | 0.976 | 0.016 | 0.003 | 2.40E-08 |
| SHBG | rs42374 | 7 | 116444070 | T | C | 0.547 | 0.006 | 0.001 | 8.60E-15 |
| SHBG | rs34748838 | 7 | 130459242 | T | C | 0.490 | 0.008 | 0.001 | 2.30E-20 |
| SHBG | rs157935 | 7 | 130585553 | G | T | 0.303 | 0.012 | 0.001 | 1.40E-39 |
| SHBG | rs200087953 | 7 | 133575017 | A | G | 0.682 | 0.005 | 0.001 | 2.60E-08 |
| SHBG | rs1559535 | 7 | 135143971 | G | A | 0.403 | 0.007 | 0.001 | 2.00E-17 |
| SHBG | rs34372369 | 7 | 143092269 | A | G | 0.052 | 0.016 | 0.002 | 2.00E-15 |
| SHBG | rs4725944 | 7 | 150476673 | C | G | 0.390 | 0.008 | 0.001 | 2.70E-21 |
| SHBG | rs114949263 | 7 | 150498245 | C | T | 0.111 | 0.015 | 0.001 | 1.70E-34 |
| SHBG | rs7808581 | 7 | 156194185 | C | T | 0.553 | 0.005 | 0.001 | 1.00E-09 |
| SHBG | rs4240624 | 8 | 9184231 | A | G | 0.908 | 0.022 | 0.001 | 2.30E-59 |
| SHBG | rs7464506 | 8 | 12624425 | A | T | 0.668 | 0.007 | 0.001 | 1.60E-13 |
| SHBG | rs113973451 | 8 | 21906789 | G | T | 0.823 | 0.007 | 0.001 | 7.70E-13 |
| SHBG | rs2241261 | 8 | 22876739 | C | T | 0.476 | 0.006 | 0.001 | 2.40E-14 |
| SHBG | rs9644032 | 8 | 23414822 | T | G | 0.367 | 0.007 | 0.001 | 7.30E-18 |
| SHBG | rs4739515 | 8 | 37391203 | C | G | 0.041 | 0.012 | 0.002 | 1.90E-08 |
| SHBG | rs117318607 | 8 | 38947274 | G | A | 0.022 | 0.015 | 0.003 | 2.50E-09 |
| SHBG | rs12543287 | 8 | 42334511 | C | G | 0.371 | 0.010 | 0.001 | 2.40E-37 |
| SHBG | rs72656017 | 8 | 57152481 | A | G | 0.870 | 0.008 | 0.001 | 6.40E-12 |
| SHBG | rs10504255 | 8 | 59398461 | A | G | 0.662 | 0.011 | 0.001 | 3.00E-36 |
| SHBG | rs72663955 | 8 | 71139330 | T | G | 0.867 | 0.007 | 0.001 | 5.30E-09 |
| SHBG | rs113605295 | 8 | 81374095 | T | C | 0.064 | 0.012 | 0.002 | 3.40E-14 |
| SHBG | rs76767219 | 8 | 81426196 | A | C | 0.035 | 0.043 | 0.002 | 6.90E-83 |
| SHBG | rs7828742 | 8 | 116960729 | A | G | 0.401 | 0.008 | 0.001 | 1.10E-20 |
| SHBG | rs11774700 | 8 | 118220270 | C | T | 0.309 | 0.006 | 0.001 | 7.30E-13 |
| SHBG | rs4871015 | 8 | 128314516 | A | G | 0.581 | 0.004 | 0.001 | 5.00E-08 |
| SHBG | rs55831924 | 8 | 145031968 | T | C | 0.361 | 0.007 | 0.001 | 3.50E-15 |
| SHBG | rs2721195 | 8 | 145677011 | T | C | 0.474 | 0.010 | 0.001 | 4.20E-33 |
| SHBG | rs1567353 | 9 | 1033773 | C | G | 0.692 | 0.008 | 0.001 | 6.60E-18 |
| SHBG | rs10757112 | 9 | 2011588 | A | G | 0.603 | 0.005 | 0.001 | 2.10E-08 |
| SHBG | rs10114763 | 9 | 4143749 | T | A | 0.423 | 0.009 | 0.001 | 3.80E-26 |
| SHBG | rs1330307 | 9 | 4305064 | A | C | 0.514 | 0.007 | 0.001 | 7.90E-18 |
| SHBG | rs79793188 | 9 | 6449701 | C | T | 0.812 | 0.006 | 0.001 | 1.00E-08 |
| SHBG | rs820504 | 9 | 6668278 | G | A | 0.864 | 0.012 | 0.001 | 1.70E-24 |
| SHBG | rs2031316 | 9 | 13563624 | A | C | 0.081 | 0.010 | 0.002 | 4.50E-10 |
| SHBG | rs10811662 | 9 | 22134253 | A | G | 0.173 | 0.007 | 0.001 | 1.10E-10 |
| SHBG | rs35234337 | 9 | 35661243 | C | T | 0.742 | 0.006 | 0.001 | 3.30E-08 |
| SHBG | rs10868080 | 9 | 86626769 | T | A | 0.256 | 0.023 | 0.001 | 3.40E-125 |
| SHBG | rs143554698 | 9 | 95538573 | C | T | 0.859 | 0.008 | 0.001 | 1.50E-12 |
| SHBG | rs1475543 | 9 | 100333671 | C | A | 0.827 | 0.006 | 0.001 | 1.40E-08 |
| SHBG | rs11515536 | 9 | 101771183 | T | C | 0.131 | 0.007 | 0.001 | 1.60E-08 |
| SHBG | rs4743776 | 9 | 107727727 | G | A | 0.299 | 0.006 | 0.001 | 1.30E-10 |
| SHBG | rs62580766 | 9 | 113034490 | T | C | 0.182 | 0.011 | 0.001 | 3.20E-27 |
| SHBG | rs6477768 | 9 | 113168239 | A | G | 0.682 | 0.006 | 0.001 | 2.50E-09 |
| SHBG | rs17372936 | 9 | 119066203 | T | C | 0.768 | 0.008 | 0.001 | 4.10E-18 |
| SHBG | rs4837794 | 9 | 123507855 | T | C | 0.331 | 0.008 | 0.001 | 1.70E-22 |
| SHBG | rs700085 | 9 | 125870466 | T | C | 0.088 | 0.009 | 0.001 | 3.70E-11 |
| SHBG | rs9697210 | 9 | 131468740 | G | A | 0.854 | 0.016 | 0.001 | 9.20E-45 |
| SHBG | rs8176693 | 9 | 136137657 | C | T | 0.938 | 0.013 | 0.002 | 7.40E-14 |
| SHBG | rs72766607 | 9 | 136895818 | T | G | 0.980 | 0.034 | 0.003 | 5.20E-32 |
| SHBG | rs11791747 | 9 | 137106879 | G | A | 0.307 | 0.007 | 0.001 | 6.90E-15 |
| SHBG | rs35233014 | 9 | 137268177 | C | A | 0.254 | 0.014 | 0.001 | 2.50E-46 |
| SHBG | rs11103377 | 9 | 139097135 | G | A | 0.539 | 0.010 | 0.001 | 1.20E-32 |
| SHBG | rs2279415 | 10 | 3792608 | G | A | 0.531 | 0.005 | 0.001 | 1.90E-08 |
| SHBG | rs79717793 | 10 | 5262267 | G | A | 0.845 | 0.021 | 0.001 | 8.50E-84 |
| SHBG | rs3824655 | 10 | 13370779 | C | G | 0.590 | 0.006 | 0.001 | 7.30E-14 |
| SHBG | rs3737178 | 10 | 31607215 | A | G | 0.951 | 0.010 | 0.002 | 1.20E-08 |
| SHBG | rs899865 | 10 | 36473044 | T | C | 0.600 | 0.005 | 0.001 | 1.20E-08 |
| SHBG | rs72783094 | 10 | 49686734 | A | G | 0.908 | 0.008 | 0.001 | 1.80E-08 |
| SHBG | rs1530439 | 10 | 63645959 | T | G | 0.309 | 0.009 | 0.001 | 7.90E-24 |
| SHBG | rs541030121 | 10 | 64871859 | A | G | 0.065 | 0.016 | 0.002 | 5.50E-15 |
| SHBG | rs537858427 | 10 | 65097399 | G | A | 0.100 | 0.010 | 0.002 | 1.50E-08 |
| SHBG | rs10822163 | 10 | 65124098 | G | C | 0.473 | 0.053 | 0.001 | 1.00E-200 |
| SHBG | rs1204083 | 10 | 69835197 | C | G | 0.346 | 0.007 | 0.001 | 2.50E-17 |
| SHBG | rs7100001 | 10 | 74654754 | A | G | 0.049 | 0.011 | 0.002 | 1.30E-08 |
| SHBG | rs2579162 | 10 | 79541639 | C | T | 0.709 | 0.004 | 0.001 | 1.50E-08 |
| SHBG | rs1782652 | 10 | 81074125 | T | A | 0.619 | 0.012 | 0.001 | 2.90E-50 |
| SHBG | rs11202594 | 10 | 89641222 | G | A | 0.843 | 0.006 | 0.001 | 9.40E-09 |
| SHBG | rs1772189 | 10 | 93629499 | T | A | 0.523 | 0.012 | 0.001 | 9.40E-54 |
| SHBG | rs2068888 | 10 | 94839642 | A | G | 0.450 | 0.011 | 0.001 | 1.70E-40 |
| SHBG | rs7080472 | 10 | 96012950 | T | G | 0.423 | 0.005 | 0.001 | 1.30E-08 |
| SHBG | rs11188601 | 10 | 97856899 | C | T | 0.364 | 0.008 | 0.001 | 1.20E-22 |
| SHBG | rs2862954 | 10 | 101912064 | C | T | 0.500 | 0.010 | 0.001 | 3.30E-28 |
| SHBG | rs67477288 | 10 | 103587858 | C | T | 0.942 | 0.010 | 0.002 | 2.70E-08 |
| SHBG | rs80235628 | 10 | 122859270 | G | A | 0.950 | 0.018 | 0.002 | 2.30E-21 |
| SHBG | rs7893136 | 10 | 122926460 | T | C | 0.021 | 0.018 | 0.003 | 5.50E-10 |
| SHBG | rs4758639 | 11 | 305406 | A | G | 0.664 | 0.007 | 0.001 | 1.00E-13 |
| SHBG | rs2412138 | 11 | 3090126 | T | C | 0.606 | 0.006 | 0.001 | 5.30E-13 |
| SHBG | rs11601507 | 11 | 5701074 | A | C | 0.069 | 0.016 | 0.002 | 1.10E-25 |
| SHBG | rs1037169 | 11 | 13361005 | T | C | 0.313 | 0.012 | 0.001 | 1.20E-41 |
| SHBG | rs2074310 | 11 | 17421886 | C | T | 0.643 | 0.006 | 0.001 | 1.50E-12 |
| SHBG | rs62618693 | 11 | 32956492 | T | C | 0.045 | 0.016 | 0.002 | 2.30E-16 |
| SHBG | rs4756190 | 11 | 35124040 | T | C | 0.580 | 0.004 | 0.001 | 1.00E-08 |
| SHBG | rs2292910 | 11 | 45903613 | A | C | 0.332 | 0.006 | 0.001 | 1.20E-11 |
| SHBG | rs566217606 | 11 | 48877002 | C | T | 0.013 | 0.022 | 0.004 | 1.40E-08 |
| SHBG | rs143709973 | 11 | 59572669 | A | C | 0.955 | 0.012 | 0.002 | 4.40E-10 |
| SHBG | rs174533 | 11 | 61549025 | G | A | 0.652 | 0.010 | 0.001 | 1.20E-28 |
| SHBG | rs12797706 | 11 | 65561369 | A | G | 0.235 | 0.014 | 0.001 | 3.20E-41 |
| SHBG | rs4988308 | 11 | 68106842 | G | A | 0.557 | 0.005 | 0.001 | 1.20E-09 |
| SHBG | rs631695 | 11 | 69283303 | T | G | 0.416 | 0.014 | 0.001 | 6.80E-68 |
| SHBG | rs75713100 | 11 | 77517973 | T | G | 0.911 | 0.008 | 0.001 | 3.50E-09 |
| SHBG | rs12575636 | 11 | 95311260 | T | G | 0.811 | 0.010 | 0.001 | 2.60E-18 |
| SHBG | rs10895276 | 11 | 102083695 | C | T | 0.658 | 0.009 | 0.001 | 3.80E-25 |
| SHBG | rs78312641 | 11 | 118749318 | A | T | 0.128 | 0.008 | 0.001 | 2.50E-10 |
| SHBG | rs55771168 | 11 | 119070949 | C | T | 0.272 | 0.007 | 0.001 | 5.70E-15 |
| SHBG | rs2156804 | 11 | 122610326 | G | T | 0.513 | 0.005 | 0.001 | 1.20E-09 |
| SHBG | rs2369280 | 12 | 508859 | T | G | 0.737 | 0.005 | 0.001 | 3.00E-08 |
| SHBG | rs56196860 | 12 | 2908330 | A | C | 0.031 | 0.023 | 0.002 | 4.40E-24 |
| SHBG | rs76895963 | 12 | 4384844 | G | T | 0.021 | 0.073 | 0.003 | 1.20E-120 |
| SHBG | rs3782735 | 12 | 6885076 | A | G | 0.600 | 0.008 | 0.001 | 1.20E-21 |
| SHBG | rs11045171 | 12 | 20470199 | G | A | 0.198 | 0.008 | 0.001 | 1.30E-14 |
| SHBG | rs57743625 | 12 | 21367633 | G | A | 0.840 | 0.030 | 0.001 | 1.10E-159 |
| SHBG | rs2900528 | 12 | 24150441 | C | G | 0.417 | 0.006 | 0.001 | 1.50E-12 |
| SHBG | rs1391790 | 12 | 24203096 | T | C | 0.964 | 0.025 | 0.002 | 1.80E-30 |
| SHBG | rs75130744 | 12 | 25410741 | G | C | 0.929 | 0.027 | 0.002 | 7.30E-72 |
| SHBG | rs2129869 | 12 | 26457650 | A | T | 0.780 | 0.007 | 0.001 | 1.20E-12 |
| SHBG | rs34072608 | 12 | 46189284 | G | T | 0.801 | 0.007 | 0.001 | 1.60E-13 |
| SHBG | rs118080406 | 12 | 47249428 | G | A | 0.025 | 0.015 | 0.003 | 2.30E-08 |
| SHBG | rs56365029 | 12 | 49036347 | A | G | 0.978 | 0.017 | 0.003 | 1.00E-10 |
| SHBG | rs864899 | 12 | 51221127 | A | G | 0.415 | 0.012 | 0.001 | 5.20E-47 |
| SHBG | rs12818938 | 12 | 53783182 | T | G | 0.832 | 0.009 | 0.001 | 4.10E-14 |
| SHBG | rs73139029 | 12 | 62822406 | A | T | 0.859 | 0.007 | 0.001 | 2.40E-09 |
| SHBG | rs145775785 | 12 | 65902265 | T | C | 0.015 | 0.023 | 0.004 | 6.10E-10 |
| SHBG | rs2583939 | 12 | 66213521 | C | T | 0.868 | 0.008 | 0.001 | 6.40E-10 |
| SHBG | rs1042725 | 12 | 66358347 | C | T | 0.508 | 0.008 | 0.001 | 7.50E-25 |
| SHBG | rs11176664 | 12 | 67661519 | A | G | 0.495 | 0.005 | 0.001 | 3.40E-09 |
| SHBG | rs2601007 | 12 | 69979115 | G | C | 0.654 | 0.005 | 0.001 | 2.70E-10 |
| SHBG | rs374335 | 12 | 77457439 | A | G | 0.681 | 0.006 | 0.001 | 2.80E-10 |
| SHBG | rs11107124 | 12 | 93988283 | G | C | 0.711 | 0.006 | 0.001 | 2.50E-10 |
| SHBG | rs11108061 | 12 | 95857620 | T | C | 0.427 | 0.005 | 0.001 | 8.70E-09 |
| SHBG | rs11111274 | 12 | 102838128 | G | A | 0.263 | 0.010 | 0.001 | 2.50E-25 |
| SHBG | rs112725417 | 12 | 112657361 | T | C | 0.973 | 0.017 | 0.003 | 3.50E-11 |
| SHBG | rs4767327 | 12 | 115929191 | T | A | 0.618 | 0.005 | 0.001 | 7.90E-10 |
| SHBG | rs113257350 | 12 | 120381998 | C | T | 0.978 | 0.017 | 0.003 | 4.60E-10 |
| SHBG | rs2393775 | 12 | 121424574 | A | G | 0.622 | 0.015 | 0.001 | 2.00E-80 |
| SHBG | rs73214164 | 12 | 121470978 | T | C | 0.802 | 0.010 | 0.001 | 4.90E-22 |
| SHBG | rs12311848 | 12 | 124486851 | G | A | 0.334 | 0.010 | 0.001 | 1.10E-33 |
| SHBG | rs1725788 | 12 | 131608476 | G | A | 0.238 | 0.006 | 0.001 | 3.30E-09 |
| SHBG | rs749170 | 13 | 22350875 | T | C | 0.335 | 0.005 | 0.001 | 7.00E-10 |
| SHBG | rs9533843 | 13 | 44980150 | A | G | 0.466 | 0.005 | 0.001 | 1.80E-08 |
| SHBG | rs41284816 | 13 | 50655989 | T | G | 0.019 | 0.031 | 0.003 | 1.50E-25 |
| SHBG | rs3116625 | 13 | 50945251 | C | T | 0.800 | 0.007 | 0.001 | 1.10E-13 |
| SHBG | rs11843816 | 13 | 91987065 | C | T | 0.965 | 0.013 | 0.002 | 3.00E-08 |
| SHBG | rs750598 | 13 | 111028978 | A | G | 0.338 | 0.005 | 0.001 | 9.50E-09 |
| SHBG | rs373373 | 13 | 111297952 | A | C | 0.948 | 0.011 | 0.002 | 1.30E-09 |
| SHBG | rs116338429 | 13 | 114767040 | T | C | 0.171 | 0.008 | 0.001 | 2.10E-11 |
| SHBG | rs112035922 | 13 | 115047464 | C | T | 0.768 | 0.008 | 0.001 | 1.90E-18 |
| SHBG | rs2064482 | 14 | 23709315 | T | C | 0.255 | 0.012 | 0.001 | 1.50E-43 |
| SHBG | rs11621792 | 14 | 24871926 | C | T | 0.547 | 0.019 | 0.001 | 1.80E-117 |
| SHBG | rs12435790 | 14 | 35154381 | G | A | 0.078 | 0.010 | 0.002 | 5.80E-10 |
| SHBG | rs2239222 | 14 | 73011885 | G | A | 0.349 | 0.011 | 0.001 | 9.40E-35 |
| SHBG | rs13379043 | 14 | 74250126 | C | T | 0.279 | 0.010 | 0.001 | 2.60E-28 |
| SHBG | rs1005421 | 14 | 89886940 | C | T | 0.584 | 0.007 | 0.001 | 7.60E-20 |
| SHBG | rs28929474 | 14 | 94844947 | T | C | 0.020 | 0.096 | 0.003 | 1.00E-200 |
| SHBG | rs17580 | 14 | 94847262 | A | T | 0.048 | 0.026 | 0.002 | 1.90E-42 |
| SHBG | rs3742366 | 14 | 104198351 | C | T | 0.346 | 0.008 | 0.001 | 9.70E-23 |
| SHBG | rs2498786 | 14 | 105262368 | C | G | 0.384 | 0.010 | 0.001 | 1.50E-32 |
| SHBG | rs56112295 | 14 | 105877057 | T | C | 0.226 | 0.006 | 0.001 | 1.60E-10 |
| SHBG | rs28510484 | 15 | 31637569 | G | C | 0.829 | 0.008 | 0.001 | 3.80E-12 |
| SHBG | rs55800572 | 15 | 35285183 | G | C | 0.339 | 0.008 | 0.001 | 6.80E-20 |
| SHBG | rs2454352 | 15 | 36047091 | C | T | 0.239 | 0.007 | 0.001 | 1.80E-12 |
| SHBG | rs275177 | 15 | 39449003 | C | T | 0.149 | 0.008 | 0.001 | 2.70E-11 |
| SHBG | rs28790585 | 15 | 39668870 | C | T | 0.705 | 0.006 | 0.001 | 1.50E-09 |
| SHBG | rs11637681 | 15 | 40387971 | A | G | 0.723 | 0.008 | 0.001 | 2.20E-18 |
| SHBG | rs139974673 | 15 | 44027885 | T | C | 0.975 | 0.066 | 0.003 | 5.10E-147 |
| SHBG | rs1008805 | 15 | 51549599 | A | G | 0.576 | 0.005 | 0.001 | 6.10E-09 |
| SHBG | rs181598957 | 15 | 53327792 | A | G | 0.018 | 0.024 | 0.003 | 3.50E-13 |
| SHBG | rs79391862 | 15 | 53739426 | A | C | 0.986 | 0.082 | 0.004 | 2.20E-121 |
| SHBG | rs4775191 | 15 | 59926946 | A | C | 0.339 | 0.007 | 0.001 | 9.80E-16 |
| SHBG | rs339998 | 15 | 60947763 | T | C | 0.394 | 0.005 | 0.001 | 5.10E-09 |
| SHBG | rs3848125 | 15 | 61957236 | A | G | 0.577 | 0.005 | 0.001 | 1.50E-11 |
| SHBG | rs76428668 | 15 | 66839282 | T | G | 0.750 | 0.009 | 0.001 | 9.10E-24 |
| SHBG | rs8038465 | 15 | 73978337 | T | C | 0.425 | 0.005 | 0.001 | 3.00E-10 |
| SHBG | rs112500920 | 15 | 82507605 | T | C | 0.930 | 0.012 | 0.002 | 4.00E-11 |
| SHBG | rs9672839 | 15 | 93568884 | C | A | 0.395 | 0.005 | 0.001 | 6.50E-09 |
| SHBG | rs12898856 | 15 | 96227920 | C | T | 0.551 | 0.010 | 0.001 | 5.40E-36 |
| SHBG | rs56332871 | 15 | 96714816 | A | C | 0.272 | 0.035 | 0.001 | 1.00E-200 |
| SHBG | rs28469124 | 16 | 1842973 | C | G | 0.087 | 0.010 | 0.001 | 6.60E-13 |
| SHBG | rs28372698 | 16 | 3115111 | A | T | 0.406 | 0.005 | 0.001 | 6.00E-09 |
| SHBG | rs3747587 | 16 | 4674954 | G | C | 0.814 | 0.010 | 0.001 | 3.00E-21 |
| SHBG | rs720130 | 16 | 11132633 | G | T | 0.595 | 0.005 | 0.001 | 5.10E-12 |
| SHBG | rs3743588 | 16 | 11836508 | G | A | 0.715 | 0.007 | 0.001 | 9.00E-17 |
| SHBG | rs12928099 | 16 | 15150505 | A | C | 0.295 | 0.010 | 0.001 | 4.00E-29 |
| SHBG | rs41278174 | 16 | 16259596 | A | G | 0.027 | 0.013 | 0.003 | 3.00E-08 |
| SHBG | rs34050011 | 16 | 49868513 | A | C | 0.424 | 0.006 | 0.001 | 1.20E-09 |
| SHBG | rs11643656 | 16 | 51463539 | G | A | 0.825 | 0.007 | 0.001 | 5.40E-09 |
| SHBG | rs246192 | 16 | 58544295 | G | C | 0.479 | 0.007 | 0.001 | 1.40E-16 |
| SHBG | rs61733486 | 16 | 68390697 | C | T | 0.942 | 0.011 | 0.002 | 5.60E-10 |
| SHBG | rs77147683 | 16 | 71623594 | A | C | 0.233 | 0.005 | 0.001 | 8.70E-09 |
| SHBG | rs2925979 | 16 | 81534790 | C | T | 0.699 | 0.007 | 0.001 | 2.30E-16 |
| SHBG | rs4782568 | 16 | 83980529 | G | C | 0.452 | 0.013 | 0.001 | 4.30E-58 |
| SHBG | rs11641834 | 16 | 88070573 | C | T | 0.569 | 0.010 | 0.001 | 1.00E-36 |
| SHBG | rs56292801 | 16 | 88535341 | A | G | 0.273 | 0.009 | 0.001 | 1.00E-25 |
| SHBG | rs11078597 | 17 | 1618363 | C | T | 0.186 | 0.017 | 0.001 | 2.70E-61 |
| SHBG | rs9902384 | 17 | 5171746 | A | G | 0.753 | 0.006 | 0.001 | 8.20E-09 |
| SHBG | rs858519 | 17 | 7531965 | C | T | 0.557 | 0.097 | 0.001 | 1.00E-200 |
| SHBG | rs8066941 | 17 | 9588450 | T | G | 0.762 | 0.012 | 0.001 | 5.90E-40 |
| SHBG | rs17669311 | 17 | 13837051 | G | A | 0.611 | 0.009 | 0.001 | 2.10E-29 |
| SHBG | rs12937088 | 17 | 16031029 | G | A | 0.508 | 0.006 | 0.001 | 8.50E-17 |
| SHBG | rs8079418 | 17 | 17924060 | C | T | 0.612 | 0.014 | 0.001 | 3.70E-65 |
| SHBG | rs12943365 | 17 | 29680526 | G | C | 0.609 | 0.009 | 0.001 | 5.50E-30 |
| SHBG | rs17138478 | 17 | 36073320 | A | C | 0.129 | 0.009 | 0.001 | 3.30E-14 |
| SHBG | rs17616365 | 17 | 38256401 | G | A | 0.968 | 0.022 | 0.002 | 3.20E-23 |
| SHBG | rs650558 | 17 | 40721042 | C | T | 0.751 | 0.007 | 0.001 | 2.20E-14 |
| SHBG | rs10048173 | 17 | 45592266 | G | A | 0.513 | 0.015 | 0.001 | 1.50E-80 |
| SHBG | rs11655704 | 17 | 47448172 | C | T | 0.314 | 0.032 | 0.001 | 1.00E-200 |
| SHBG | rs8077316 | 17 | 48626389 | T | C | 0.246 | 0.006 | 0.001 | 1.60E-10 |
| SHBG | rs8074363 | 17 | 59241469 | C | T | 0.775 | 0.006 | 0.001 | 6.10E-11 |
| SHBG | rs76708468 | 17 | 62206299 | T | C | 0.961 | 0.013 | 0.002 | 4.50E-09 |
| SHBG | rs17650301 | 17 | 62479273 | C | A | 0.294 | 0.005 | 0.001 | 1.20E-09 |
| SHBG | rs1801689 | 17 | 64210580 | A | C | 0.970 | 0.038 | 0.002 | 3.00E-60 |
| SHBG | rs7211695 | 17 | 65235971 | A | G | 0.521 | 0.008 | 0.001 | 1.40E-24 |
| SHBG | rs34931250 | 17 | 66879927 | T | C | 0.061 | 0.010 | 0.002 | 6.10E-10 |
| SHBG | rs9282552 | 17 | 67109894 | T | C | 0.650 | 0.007 | 0.001 | 4.70E-18 |
| SHBG | rs1605750 | 17 | 68471073 | A | G | 0.510 | 0.005 | 0.001 | 4.60E-10 |
| SHBG | rs72844546 | 17 | 73149850 | C | T | 0.346 | 0.010 | 0.001 | 2.80E-33 |
| SHBG | rs2587505 | 17 | 77784268 | T | C | 0.580 | 0.006 | 0.001 | 1.30E-11 |
| SHBG | rs10153315 | 17 | 79481772 | T | C | 0.582 | 0.008 | 0.001 | 2.90E-24 |
| SHBG | rs11664106 | 18 | 2846812 | T | A | 0.373 | 0.007 | 0.001 | 7.10E-17 |
| SHBG | rs9945126 | 18 | 46469962 | C | G | 0.465 | 0.004 | 0.001 | 1.30E-08 |
| SHBG | rs55855238 | 18 | 55089715 | C | T | 0.650 | 0.010 | 0.001 | 5.10E-29 |
| SHBG | rs6567230 | 18 | 59330824 | T | A | 0.701 | 0.006 | 0.001 | 1.60E-11 |
| SHBG | rs620068 | 18 | 60140576 | A | G | 0.459 | 0.005 | 0.001 | 7.60E-10 |
| SHBG | rs12454712 | 18 | 60845884 | C | T | 0.377 | 0.010 | 0.001 | 1.00E-32 |
| SHBG | rs3829639 | 18 | 71943144 | A | G | 0.672 | 0.008 | 0.001 | 1.30E-19 |
| SHBG | rs34668346 | 19 | 672169 | A | G | 0.513 | 0.005 | 0.001 | 8.90E-10 |
| SHBG | rs150122016 | 19 | 1246079 | G | T | 0.972 | 0.016 | 0.003 | 8.10E-09 |
| SHBG | rs1640269 | 19 | 2793194 | C | A | 0.286 | 0.019 | 0.001 | 9.10E-101 |
| SHBG | rs11539938 | 19 | 3062857 | C | T | 0.421 | 0.010 | 0.001 | 2.40E-29 |
| SHBG | rs60018147 | 19 | 3375572 | G | A | 0.120 | 0.014 | 0.001 | 2.60E-25 |
| SHBG | rs10221473 | 19 | 7236626 | G | A | 0.542 | 0.007 | 0.001 | 6.60E-13 |
| SHBG | rs8107967 | 19 | 7972615 | G | A | 0.567 | 0.007 | 0.001 | 1.30E-15 |
| SHBG | rs281439 | 19 | 10400110 | C | G | 0.780 | 0.006 | 0.001 | 3.00E-08 |
| SHBG | rs8101895 | 19 | 12508061 | A | T | 0.236 | 0.007 | 0.001 | 3.50E-13 |
| SHBG | rs7252372 | 19 | 14172896 | G | C | 0.556 | 0.009 | 0.001 | 8.20E-27 |
| SHBG | rs202200760 | 19 | 17346854 | C | G | 0.039 | 0.073 | 0.002 | 1.00E-200 |
| SHBG | rs4805881 | 19 | 33896432 | C | A | 0.666 | 0.009 | 0.001 | 4.30E-29 |
| SHBG | rs45512696 | 19 | 35550878 | T | C | 0.175 | 0.021 | 0.001 | 2.00E-86 |
| SHBG | rs11666245 | 19 | 38229926 | G | A | 0.953 | 0.017 | 0.002 | 7.00E-20 |
| SHBG | rs11673023 | 19 | 38239521 | C | A | 0.829 | 0.007 | 0.001 | 9.60E-12 |
| SHBG | rs483082 | 19 | 45416178 | T | G | 0.237 | 0.009 | 0.001 | 1.90E-21 |
| SHBG | rs5112 | 19 | 45430280 | G | C | 0.533 | 0.008 | 0.001 | 4.90E-20 |
| SHBG | rs34255979 | 19 | 46384830 | T | C | 0.120 | 0.028 | 0.001 | 1.50E-108 |
| SHBG | rs111981233 | 19 | 50016479 | G | T | 0.080 | 0.021 | 0.002 | 1.60E-46 |
| SHBG | rs11672485 | 19 | 53833712 | C | T | 0.490 | 0.005 | 0.001 | 1.10E-12 |
| SHBG | rs4077285 | 19 | 56599405 | G | C | 0.906 | 0.012 | 0.001 | 1.90E-15 |
| SHBG | rs16988208 | 19 | 58332315 | G | T | 0.164 | 0.007 | 0.001 | 1.70E-10 |
| SHBG | rs11545185 | 19 | 59028585 | A | G | 0.171 | 0.009 | 0.001 | 2.20E-16 |
| SHBG | rs144033177 | 20 | 571467 | A | C | 0.984 | 0.018 | 0.003 | 1.10E-08 |
| SHBG | rs1741288 | 20 | 4102954 | A | G | 0.635 | 0.005 | 0.001 | 2.30E-11 |
| SHBG | rs7261425 | 20 | 20068635 | G | C | 0.278 | 0.005 | 0.001 | 2.70E-08 |
| SHBG | rs6048205 | 20 | 22559601 | A | G | 0.957 | 0.012 | 0.002 | 3.60E-08 |
| SHBG | rs13042148 | 20 | 32298286 | C | T | 0.845 | 0.014 | 0.001 | 9.00E-39 |
| SHBG | rs6029640 | 20 | 39970385 | G | A | 0.420 | 0.010 | 0.001 | 9.60E-33 |
| SHBG | rs6073431 | 20 | 43040569 | T | C | 0.531 | 0.017 | 0.001 | 5.10E-92 |
| SHBG | rs1412957 | 20 | 45557065 | A | G | 0.574 | 0.007 | 0.001 | 3.50E-16 |
| SHBG | rs6018424 | 20 | 45986984 | T | C | 0.199 | 0.008 | 0.001 | 4.40E-14 |
| SHBG | rs55987409 | 20 | 49569025 | T | C | 0.072 | 0.018 | 0.002 | 6.40E-28 |
| SHBG | rs61744628 | 20 | 52186837 | A | G | 0.027 | 0.017 | 0.003 | 1.70E-11 |
| SHBG | rs6123685 | 20 | 55836040 | A | G | 0.254 | 0.005 | 0.001 | 3.80E-09 |
| SHBG | rs62217799 | 20 | 62347191 | G | T | 0.342 | 0.007 | 0.001 | 2.20E-15 |
| SHBG | rs1475883 | 21 | 17591913 | G | A | 0.567 | 0.004 | 0.001 | 2.40E-08 |
| SHBG | rs112078975 | 21 | 33095959 | G | A | 0.951 | 0.012 | 0.002 | 8.40E-11 |
| SHBG | rs4818008 | 21 | 40611442 | T | A | 0.645 | 0.005 | 0.001 | 2.70E-08 |
| SHBG | rs35598889 | 22 | 18450794 | C | T | 0.233 | 0.007 | 0.001 | 8.50E-15 |
| SHBG | rs759404 | 22 | 18916180 | C | T | 0.932 | 0.010 | 0.002 | 4.00E-08 |
| SHBG | rs9606233 | 22 | 20066611 | C | G | 0.405 | 0.005 | 0.001 | 8.90E-12 |
| SHBG | rs4820091 | 22 | 21940189 | G | T | 0.181 | 0.011 | 0.001 | 1.60E-21 |
| SHBG | rs6005840 | 22 | 29101357 | A | G | 0.326 | 0.012 | 0.001 | 3.00E-42 |
| SHBG | rs5749082 | 22 | 30770603 | T | A | 0.292 | 0.011 | 0.001 | 3.10E-36 |
| SHBG | rs9610329 | 22 | 36042986 | C | T | 0.572 | 0.006 | 0.001 | 7.60E-13 |
| SHBG | rs13057133 | 22 | 38179473 | C | T | 0.699 | 0.007 | 0.001 | 3.00E-15 |
| SHBG | rs2075915 | 22 | 39254556 | A | G | 0.747 | 0.006 | 0.001 | 2.40E-11 |
| SHBG | rs738409 | 22 | 44324727 | G | C | 0.216 | 0.023 | 0.001 | 3.10E-114 |
| SHBG | rs135563 | 22 | 46536017 | T | C | 0.510 | 0.005 | 0.001 | 1.70E-09 |
| SHBG | rs36171610 | 22 | 50422385 | G | A | 0.509 | 0.006 | 0.001 | 2.00E-11 |
| Testosterone | rs182050989 | 1 | 27262545 | C | T | 0.971 | 0.027 | 0.004 | 1.20E-12 |
| Testosterone | rs4453027 | 1 | 41453453 | G | T | 0.575 | 0.010 | 0.001 | 5.50E-15 |
| Testosterone | rs4912377 | 1 | 57015121 | C | A | 0.487 | 0.008 | 0.001 | 1.40E-09 |
| Testosterone | rs12745935 | 1 | 59644082 | G | A | 0.300 | 0.008 | 0.001 | 1.50E-08 |
| Testosterone | rs6684361 | 1 | 101737743 | C | T | 0.308 | 0.025 | 0.001 | 2.50E-70 |
| Testosterone | rs1977658 | 1 | 107607037 | T | G | 0.344 | 0.015 | 0.001 | 1.60E-27 |
| Testosterone | rs41264630 | 1 | 149885800 | A | G | 0.080 | 0.014 | 0.002 | 3.90E-09 |
| Testosterone | rs267733 | 1 | 150958836 | A | G | 0.839 | 0.012 | 0.002 | 1.20E-12 |
| Testosterone | rs1870940 | 1 | 154984363 | G | A | 0.728 | 0.010 | 0.001 | 2.90E-12 |
| Testosterone | rs72708239 | 1 | 155985899 | A | G | 0.739 | 0.010 | 0.001 | 1.50E-10 |
| Testosterone | rs733190 | 1 | 172095226 | T | C | 0.526 | 0.008 | 0.001 | 1.60E-09 |
| Testosterone | rs61320678 | 1 | 214177319 | G | T | 0.236 | 0.010 | 0.002 | 2.30E-11 |
| Testosterone | rs78851238 | 1 | 216876964 | C | T | 0.172 | 0.009 | 0.002 | 5.00E-08 |
| Testosterone | rs61823391 | 1 | 218541992 | T | C | 0.683 | 0.011 | 0.001 | 4.20E-16 |
| Testosterone | rs62115715 | 2 | 12531863 | C | T | 0.028 | 0.030 | 0.004 | 8.30E-14 |
| Testosterone | rs3771243 | 2 | 20412227 | A | G | 0.389 | 0.009 | 0.001 | 2.50E-12 |
| Testosterone | rs1260326 | 2 | 27730940 | C | T | 0.606 | 0.028 | 0.001 | 3.50E-103 |
| Testosterone | rs2374456 | 2 | 43271621 | G | C | 0.585 | 0.008 | 0.001 | 1.00E-09 |
| Testosterone | rs58839393 | 2 | 43490619 | A | T | 0.839 | 0.016 | 0.002 | 3.90E-19 |
| Testosterone | rs111861797 | 2 | 48022688 | T | C | 0.807 | 0.012 | 0.002 | 3.30E-13 |
| Testosterone | rs11888201 | 2 | 60066405 | C | G | 0.542 | 0.007 | 0.001 | 3.30E-08 |
| Testosterone | rs2723065 | 2 | 65279414 | G | A | 0.375 | 0.011 | 0.001 | 1.30E-14 |
| Testosterone | rs10202148 | 2 | 86163767 | G | A | 0.739 | 0.009 | 0.001 | 2.70E-10 |
| Testosterone | rs590097 | 2 | 111934107 | G | T | 0.647 | 0.019 | 0.001 | 3.80E-48 |
| Testosterone | rs62162863 | 2 | 112071459 | G | T | 0.577 | 0.010 | 0.001 | 6.60E-11 |
| Testosterone | rs1128249 | 2 | 165528624 | T | G | 0.393 | 0.009 | 0.001 | 4.10E-13 |
| Testosterone | rs56117787 | 2 | 178154343 | G | A | 0.199 | 0.013 | 0.002 | 1.90E-14 |
| Testosterone | rs2011425 | 2 | 234627608 | T | G | 0.920 | 0.025 | 0.002 | 3.10E-26 |
| Testosterone | rs7618363 | 3 | 10545125 | C | G | 0.841 | 0.012 | 0.002 | 1.30E-10 |
| Testosterone | rs3103310 | 3 | 12473045 | A | G | 0.758 | 0.009 | 0.002 | 2.70E-09 |
| Testosterone | rs73075656 | 3 | 41105947 | G | T | 0.867 | 0.015 | 0.002 | 2.10E-15 |
| Testosterone | rs57615517 | 3 | 52322417 | G | A | 0.685 | 0.008 | 0.001 | 4.70E-10 |
| Testosterone | rs696516 | 3 | 136067778 | G | T | 0.266 | 0.008 | 0.001 | 1.10E-09 |
| Testosterone | rs4678408 | 3 | 138053187 | G | A | 0.629 | 0.009 | 0.001 | 5.90E-12 |
| Testosterone | rs9850919 | 3 | 169177924 | C | T | 0.406 | 0.007 | 0.001 | 4.70E-08 |
| Testosterone | rs56271032 | 3 | 172154210 | G | A | 0.131 | 0.011 | 0.002 | 3.60E-09 |
| Testosterone | rs36205397 | 4 | 3470604 | G | A | 0.421 | 0.010 | 0.001 | 1.30E-13 |
| Testosterone | rs7686914 | 4 | 69537915 | C | T | 0.475 | 0.011 | 0.001 | 9.20E-18 |
| Testosterone | rs4632729 | 4 | 69946004 | A | G | 0.545 | 0.015 | 0.001 | 2.10E-30 |
| Testosterone | rs13152154 | 4 | 77417756 | C | T | 0.273 | 0.008 | 0.001 | 7.30E-09 |
| Testosterone | rs1408 | 4 | 88057353 | G | A | 0.422 | 0.011 | 0.001 | 6.80E-19 |
| Testosterone | rs10017280 | 4 | 104609388 | C | T | 0.870 | 0.012 | 0.002 | 1.20E-09 |
| Testosterone | rs2903385 | 4 | 106094427 | A | G | 0.485 | 0.009 | 0.001 | 2.10E-14 |
| Testosterone | rs4245930 | 4 | 109038654 | G | A | 0.367 | 0.007 | 0.001 | 2.40E-08 |
| Testosterone | rs62314881 | 4 | 113956403 | G | T | 0.817 | 0.011 | 0.002 | 2.10E-10 |
| Testosterone | rs11937496 | 4 | 149613443 | G | A | 0.556 | 0.007 | 0.001 | 2.90E-08 |
| Testosterone | rs112694713 | 5 | 35247932 | A | G | 0.987 | 0.041 | 0.006 | 3.50E-13 |
| Testosterone | rs9686661 | 5 | 55861786 | C | T | 0.800 | 0.012 | 0.002 | 1.30E-14 |
| Testosterone | rs4431325 | 5 | 76461706 | T | C | 0.059 | 0.021 | 0.003 | 9.90E-15 |
| Testosterone | rs77741622 | 5 | 76491857 | G | A | 0.653 | 0.009 | 0.001 | 9.20E-11 |
| Testosterone | rs784420 | 5 | 77987524 | G | A | 0.288 | 0.013 | 0.001 | 3.20E-21 |
| Testosterone | rs112530420 | 5 | 95871370 | C | T | 0.166 | 0.010 | 0.002 | 2.30E-08 |
| Testosterone | rs12658172 | 5 | 124205385 | G | C | 0.842 | 0.018 | 0.002 | 2.70E-23 |
| Testosterone | rs4835948 | 5 | 127870190 | T | C | 0.533 | 0.007 | 0.001 | 2.90E-09 |
| Testosterone | rs13185520 | 5 | 137807260 | A | G | 0.575 | 0.008 | 0.001 | 9.10E-10 |
| Testosterone | rs62394296 | 6 | 25870381 | T | C | 0.117 | 0.012 | 0.002 | 4.20E-10 |
| Testosterone | rs9272309 | 6 | 32603936 | A | G | 0.620 | 0.013 | 0.001 | 1.10E-19 |
| Testosterone | rs6939861 | 6 | 41703041 | G | A | 0.738 | 0.009 | 0.001 | 3.40E-09 |
| Testosterone | rs6458331 | 6 | 43280028 | T | C | 0.725 | 0.014 | 0.001 | 1.20E-22 |
| Testosterone | rs57323441 | 6 | 52404343 | C | A | 0.123 | 0.011 | 0.002 | 2.20E-09 |
| Testosterone | rs1681967 | 6 | 64270790 | A | G | 0.918 | 0.015 | 0.002 | 5.90E-10 |
| Testosterone | rs221584 | 6 | 99739942 | G | A | 0.817 | 0.010 | 0.002 | 1.60E-08 |
| Testosterone | rs7759938 | 6 | 105378954 | T | C | 0.678 | 0.010 | 0.001 | 1.70E-13 |
| Testosterone | rs36182456 | 6 | 119167949 | A | G | 0.779 | 0.018 | 0.002 | 2.10E-33 |
| Testosterone | rs577721086 | 6 | 127440047 | T | C | 0.950 | 0.020 | 0.003 | 3.40E-11 |
| Testosterone | rs520829 | 6 | 160767905 | G | T | 0.475 | 0.010 | 0.001 | 2.20E-16 |
| Testosterone | rs12702516 | 7 | 6526006 | A | C | 0.152 | 0.011 | 0.002 | 3.70E-09 |
| Testosterone | rs10278686 | 7 | 15031450 | T | C | 0.491 | 0.010 | 0.001 | 1.30E-14 |
| Testosterone | rs6462989 | 7 | 40869754 | A | C | 0.659 | 0.008 | 0.001 | 1.50E-09 |
| Testosterone | rs34060476 | 7 | 73037956 | G | A | 0.134 | 0.020 | 0.002 | 5.30E-26 |
| Testosterone | rs445 | 7 | 92408370 | C | T | 0.905 | 0.012 | 0.002 | 1.20E-08 |
| Testosterone | rs45446698 | 7 | 99332948 | T | G | 0.958 | 0.120 | 0.003 | 1.00E-200 |
| Testosterone | rs11556924 | 7 | 129663496 | T | C | 0.389 | 0.008 | 0.001 | 1.20E-09 |
| Testosterone | rs157934 | 7 | 130585492 | C | T | 0.305 | 0.008 | 0.001 | 3.40E-09 |
| Testosterone | rs2306847 | 7 | 137798593 | T | A | 0.791 | 0.019 | 0.002 | 5.20E-34 |
| Testosterone | rs4725944 | 7 | 150476673 | C | G | 0.390 | 0.007 | 0.001 | 4.60E-08 |
| Testosterone | rs9638084 | 7 | 156311745 | G | A | 0.604 | 0.007 | 0.001 | 1.60E-08 |
| Testosterone | rs73200740 | 8 | 10627311 | A | C | 0.236 | 0.009 | 0.002 | 1.80E-09 |
| Testosterone | rs201643157 | 8 | 23399890 | T | C | 0.250 | 0.009 | 0.001 | 9.00E-10 |
| Testosterone | rs881301 | 8 | 38332318 | T | C | 0.587 | 0.008 | 0.001 | 6.40E-10 |
| Testosterone | rs35222808 | 8 | 77879704 | C | A | 0.748 | 0.009 | 0.001 | 5.70E-10 |
| Testosterone | rs150539196 | 8 | 81399180 | G | A | 0.036 | 0.022 | 0.004 | 3.50E-10 |
| Testosterone | rs34955534 | 8 | 81710349 | G | A | 0.901 | 0.015 | 0.002 | 6.50E-12 |
| Testosterone | rs35783704 | 8 | 105966258 | A | G | 0.101 | 0.020 | 0.002 | 2.60E-20 |
| Testosterone | rs6471583 | 8 | 143991092 | G | A | 0.443 | 0.011 | 0.001 | 1.10E-19 |
| Testosterone | rs12683780 | 9 | 16252807 | A | C | 0.669 | 0.010 | 0.001 | 2.80E-13 |
| Testosterone | rs4961485 | 9 | 16360889 | T | C | 0.933 | 0.016 | 0.003 | 5.90E-10 |
| Testosterone | rs17810415 | 9 | 19032907 | G | A | 0.200 | 0.010 | 0.002 | 6.70E-09 |
| Testosterone | rs10971921 | 9 | 34111002 | A | G | 0.131 | 0.014 | 0.002 | 7.40E-13 |
| Testosterone | rs1547308 | 9 | 114603240 | C | T | 0.800 | 0.012 | 0.002 | 6.80E-15 |
| Testosterone | rs10817260 | 9 | 114828332 | C | T | 0.810 | 0.014 | 0.002 | 6.90E-17 |
| Testosterone | rs10982192 | 9 | 117149417 | T | C | 0.221 | 0.009 | 0.002 | 2.20E-08 |
| Testosterone | rs494242 | 9 | 136145118 | C | T | 0.660 | 0.008 | 0.001 | 3.10E-09 |
| Testosterone | rs35182096 | 9 | 137268682 | C | T | 0.256 | 0.008 | 0.001 | 3.60E-08 |
| Testosterone | rs36032941 | 10 | 5062752 | C | A | 0.704 | 0.023 | 0.001 | 5.00E-56 |
| Testosterone | rs1171617 | 10 | 61467182 | T | G | 0.767 | 0.017 | 0.002 | 8.50E-31 |
| Testosterone | rs2675611 | 10 | 63644246 | T | C | 0.469 | 0.007 | 0.001 | 3.40E-08 |
| Testosterone | rs10740131 | 10 | 65271488 | T | A | 0.473 | 0.038 | 0.001 | 2.50E-196 |
| Testosterone | rs1204083 | 10 | 69835197 | C | G | 0.346 | 0.012 | 0.001 | 2.80E-18 |
| Testosterone | rs1782652 | 10 | 81074125 | T | A | 0.619 | 0.008 | 0.001 | 9.50E-09 |
| Testosterone | rs11572082 | 10 | 96826922 | C | G | 0.878 | 0.013 | 0.002 | 8.00E-11 |
| Testosterone | rs11191801 | 10 | 105532165 | A | C | 0.707 | 0.009 | 0.001 | 6.00E-12 |
| Testosterone | rs74440003 | 10 | 111995456 | A | T | 0.261 | 0.008 | 0.001 | 1.10E-08 |
| Testosterone | rs11564722 | 11 | 2178330 | T | C | 0.240 | 0.009 | 0.002 | 8.20E-09 |
| Testosterone | rs7937758 | 11 | 10236478 | A | G | 0.504 | 0.007 | 0.001 | 2.90E-08 |
| Testosterone | rs11023881 | 11 | 16246700 | T | A | 0.612 | 0.009 | 0.001 | 1.70E-11 |
| Testosterone | rs2035838 | 11 | 29200384 | A | G | 0.852 | 0.011 | 0.002 | 2.50E-10 |
| Testosterone | rs12294104 | 11 | 30382899 | T | C | 0.173 | 0.009 | 0.002 | 9.40E-09 |
| Testosterone | rs171021 | 11 | 72317557 | C | T | 0.703 | 0.014 | 0.001 | 2.70E-21 |
| Testosterone | rs12796488 | 11 | 94131557 | C | A | 0.824 | 0.010 | 0.002 | 1.40E-08 |
| Testosterone | rs4938576 | 11 | 118746769 | G | T | 0.588 | 0.007 | 0.001 | 1.50E-08 |
| Testosterone | rs10892924 | 11 | 122773715 | T | A | 0.568 | 0.011 | 0.001 | 2.70E-17 |
| Testosterone | rs12788072 | 11 | 123334298 | G | A | 0.178 | 0.010 | 0.002 | 2.70E-08 |
| Testosterone | rs618888 | 11 | 125081521 | T | G | 0.283 | 0.008 | 0.001 | 3.50E-08 |
| Testosterone | rs56196860 | 12 | 2908330 | A | C | 0.031 | 0.064 | 0.004 | 7.20E-71 |
| Testosterone | rs180435 | 12 | 47180370 | G | C | 0.809 | 0.010 | 0.002 | 3.00E-09 |
| Testosterone | rs2583949 | 12 | 66194243 | C | T | 0.895 | 0.012 | 0.002 | 5.00E-09 |
| Testosterone | rs9509847 | 13 | 22318314 | C | A | 0.628 | 0.020 | 0.001 | 1.60E-52 |
| Testosterone | rs41284816 | 13 | 50655989 | T | G | 0.019 | 0.026 | 0.005 | 1.50E-08 |
| Testosterone | rs9543012 | 13 | 73172648 | C | T | 0.353 | 0.008 | 0.001 | 2.60E-10 |
| Testosterone | rs2038695 | 13 | 100559123 | C | A | 0.449 | 0.007 | 0.001 | 2.10E-08 |
| Testosterone | rs72660136 | 13 | 109960307 | T | C | 0.966 | 0.021 | 0.004 | 8.50E-10 |
| Testosterone | rs7342537 | 14 | 21555063 | G | A | 0.018 | 0.036 | 0.005 | 5.50E-13 |
| Testosterone | rs2256191 | 14 | 64656855 | T | C | 0.655 | 0.008 | 0.001 | 7.40E-09 |
| Testosterone | rs28929474 | 14 | 94844947 | T | C | 0.020 | 0.040 | 0.005 | 1.10E-17 |
| Testosterone | rs12436785 | 14 | 98550490 | C | T | 0.416 | 0.011 | 0.001 | 7.00E-16 |
| Testosterone | rs28576256 | 14 | 99718173 | G | A | 0.869 | 0.013 | 0.002 | 9.10E-12 |
| Testosterone | rs11629457 | 14 | 100805583 | T | C | 0.265 | 0.008 | 0.001 | 1.20E-08 |
| Testosterone | rs73365510 | 14 | 106875035 | A | G | 0.071 | 0.022 | 0.003 | 4.00E-12 |
| Testosterone | rs7183977 | 15 | 40377092 | C | T | 0.347 | 0.021 | 0.001 | 3.10E-57 |
| Testosterone | rs55707100 | 15 | 43820717 | C | T | 0.975 | 0.027 | 0.004 | 7.70E-12 |
| Testosterone | rs79391862 | 15 | 53739426 | A | C | 0.986 | 0.041 | 0.006 | 2.40E-14 |
| Testosterone | rs35698268 | 15 | 60978246 | G | C | 0.224 | 0.010 | 0.002 | 4.60E-10 |
| Testosterone | rs12914034 | 15 | 65941010 | A | T | 0.637 | 0.007 | 0.001 | 4.70E-08 |
| Testosterone | rs2201003 | 15 | 75463144 | A | G | 0.364 | 0.008 | 0.001 | 6.70E-10 |
| Testosterone | rs4464040 | 15 | 79840557 | C | T | 0.850 | 0.014 | 0.002 | 2.80E-16 |
| Testosterone | rs35816571 | 15 | 85554122 | C | G | 0.829 | 0.014 | 0.002 | 1.40E-16 |
| Testosterone | rs1822246 | 15 | 96245020 | A | G | 0.703 | 0.008 | 0.001 | 1.60E-09 |
| Testosterone | rs56332871 | 15 | 96714816 | A | C | 0.273 | 0.017 | 0.001 | 3.50E-34 |
| Testosterone | rs370222 | 16 | 4157696 | G | A | 0.697 | 0.010 | 0.001 | 3.10E-12 |
| Testosterone | rs28495625 | 16 | 11891361 | A | C | 0.149 | 0.013 | 0.002 | 1.20E-12 |
| Testosterone | rs2764772 | 16 | 20060653 | A | T | 0.334 | 0.008 | 0.001 | 9.90E-10 |
| Testosterone | rs2061679 | 16 | 81573976 | T | C | 0.066 | 0.033 | 0.003 | 1.50E-36 |
| Testosterone | rs2287322 | 17 | 1641035 | G | A | 0.222 | 0.009 | 0.002 | 1.10E-08 |
| Testosterone | rs1799941 | 17 | 7533423 | A | G | 0.261 | 0.064 | 0.001 | 1.00E-200 |
| Testosterone | rs941446 | 17 | 17680273 | T | C | 0.632 | 0.008 | 0.001 | 3.50E-10 |
| Testosterone | rs55885610 | 17 | 27579011 | C | T | 0.964 | 0.023 | 0.004 | 2.60E-10 |
| Testosterone | rs9913470 | 17 | 29566575 | A | G | 0.803 | 0.014 | 0.002 | 5.30E-15 |
| Testosterone | rs1317701 | 17 | 47444794 | C | T | 0.312 | 0.017 | 0.001 | 3.70E-34 |
| Testosterone | rs28421540 | 18 | 3818842 | A | C | 0.715 | 0.011 | 0.001 | 3.00E-14 |
| Testosterone | rs17089026 | 18 | 71936165 | G | T | 0.023 | 0.053 | 0.004 | 7.10E-37 |
| Testosterone | rs79384925 | 18 | 71938618 | C | A | 0.863 | 0.026 | 0.002 | 2.00E-41 |
| Testosterone | rs759068 | 19 | 2792566 | A | G | 0.294 | 0.011 | 0.001 | 5.20E-16 |
| Testosterone | rs77017252 | 19 | 7218922 | T | A | 0.756 | 0.010 | 0.002 | 4.80E-12 |
| Testosterone | rs8111359 | 19 | 10471462 | C | T | 0.905 | 0.020 | 0.002 | 1.90E-19 |
| Testosterone | rs4804669 | 19 | 12502457 | A | G | 0.217 | 0.013 | 0.002 | 2.40E-17 |
| Testosterone | rs146497684 | 19 | 17346441 | T | A | 0.030 | 0.044 | 0.004 | 1.20E-29 |
| Testosterone | rs34858588 | 19 | 19457235 | C | G | 0.921 | 0.016 | 0.002 | 4.30E-10 |
| Testosterone | rs11673591 | 19 | 41985931 | A | T | 0.252 | 0.012 | 0.001 | 4.80E-17 |
| Testosterone | rs67967246 | 19 | 46408018 | C | A | 0.132 | 0.015 | 0.002 | 9.20E-16 |
| Testosterone | rs2241388 | 19 | 47572987 | T | C | 0.279 | 0.011 | 0.001 | 3.20E-14 |
| Testosterone | rs78248023 | 19 | 49515171 | A | C | 0.078 | 0.019 | 0.002 | 1.60E-16 |
| Testosterone | rs6130613 | 20 | 43054441 | T | C | 0.470 | 0.011 | 0.001 | 2.90E-15 |
| Testosterone | rs6020423 | 20 | 48909667 | C | T | 0.760 | 0.010 | 0.002 | 6.40E-12 |
| Testosterone | rs2273991 | 20 | 60588049 | G | C | 0.897 | 0.013 | 0.002 | 3.00E-09 |
| Testosterone | rs8126001 | 20 | 62711459 | T | C | 0.490 | 0.010 | 0.001 | 2.30E-14 |
| Testosterone | rs12185851 | 21 | 43372219 | C | T | 0.232 | 0.009 | 0.002 | 6.70E-09 |
| Testosterone | rs5752773 | 22 | 29105415 | G | C | 0.327 | 0.011 | 0.001 | 2.20E-14 |
| Testosterone | rs5763800 | 22 | 30533409 | A | G | 0.977 | 0.027 | 0.004 | 9.00E-10 |
| Testosterone | rs5751229 | 22 | 42545221 | A | G | 0.228 | 0.011 | 0.002 | 1.50E-12 |
| Testosterone | rs738409 | 22 | 44324727 | G | C | 0.216 | 0.015 | 0.002 | 7.40E-21 |
| Testosterone | rs41378347 | 22 | 46636976 | G | A | 0.888 | 0.015 | 0.002 | 5.20E-14 |
| Testosterone | rs62220604 | 22 | 49677464 | A | G | 0.283 | 0.008 | 0.001 | 1.60E-09 |

EAF, effect allele frequency; SE, standard error; SHBG, sex hormone binding globulin.

**Supplementary Table 2.** Information on genome-wide analysis on sex hormones in the UK Biobank study

| **Hormone** | **Sex** | **Description of phenotype** | **Units** | **Inclusions/exclusions** | **Transformation** | **Covariates** | **N** |
| --- | --- | --- | --- | --- | --- | --- | --- |
| Estradiol in men | Men | Binary phenotype based on an indication of measurable estradiol (UK Biobank variable 30800) compared with those indicated as below lower limit (UK Biobank variable 30806=4 or 2). | Binary | Men who self-reported taking hormone-based medication (UK Biobank variables 30850 and 20003). Included white Europeans only. | NA | Dichotomous - men at lower limit vs others. Age covariate | 206 927 |
| Total testosterone in men and women combined | Women and men | Testosterone (UK Biobank variable 30850) including people where testosterone was not reportable as below lower limit (UK Biobank variable 30856=4). | nmol/L | Participants with missing values that were not reportable at assay as too low were set to 0.3, a value below the lower limit of detection. Included white Europeans only. | Inverse normal transformation of rank | Fasting time, age, center, chip/release of genetic data | 425 097 |
| SHBG adjusted for BMI in men and women combined | Women and men | SHBG (UK Biobank variable 30830). Adjusted for body mass index. | nmol/L | Women or men who self-reported taking hormone-based medication including HRT and oral contraception at the time of the initial visit (UK Biobank variables 30850 and 20003). Included white Europeans only. | Revisualized natural log calculated separately in men and women | Men: Age, batch, body mass index, dilution. Women: Age, body mass index, batch, dilution, menopause status, and operation status | 368 929 |

SHBG, sex hormone binding globulin**.**

**Supplementary Table 3**. Information on used consortia and studies in Mendelian randomization analysis

| **Outcome** | **Study or consortium** | **Data source** | **Population** | **Cases** | **Controls** |
| --- | --- | --- | --- | --- | --- |
| Hypertension | FinnGen | <https://r5.finngen.fi/pheno/I9_HYPTENS> | European | 55,917 | 162,837 |
| Hypertensive heart disease | FinnGen | <https://r5.finngen.fi/pheno/I9_HYPTENSHD> | European | 3938 | 162,837 |
| Hypertensive renal disease | FinnGen | <https://r5.finngen.fi/pheno/I9_HYPTENSRD> | European | 468 | 162,837 |
| Type 2 diabetes | DIAGRAM | <https://www.diagram-consortium.org/downloads.html> | European | 74,124 | 824,006 |
|  | FinnGen | <https://r5.finngen.fi/pheno/E4_DM2_STRICT> | European | 29,166 | 183,185 |
| Diabetic ophthalmic complications | FinnGen | <https://r5.finngen.fi/pheno/E4_DM2OPTH> | European | 2119 | 183,185 |
| Diabetic neuropathy | FinnGen | <https://r5.finngen.fi/pheno/DM_NEUROPATHY> | European | 1415 | 162,201 |
| Coronary artery disease | CARDIoGRAMplusC4D plus UKBB | MR-Base: ebi-a-GCST005195 | Mixed | 122,733 | 424,528 |
|  | FinnGen | <https://r5.finngen.fi/pheno/I9_ISCHHEART> | European | 30,952 | 187,840 |
| Myocardial infarction | CARDIoGRAMplusC4D plus UKBB | <https://www.ebi.ac.uk/gwas/publications/33532862> | European | 61,505 | 577,716 |
|  | FinnGen | <https://r5.finngen.fi/pheno/I9_MI_STRICT> | European | 11,622 | 187,840 |
| Angina pectoris | FinnGen | <https://r5.finngen.fi/pheno/I9_ANGINA> | European | 18,168 | 187,840 |
| Unstable angina | FinnGen | <https://r5.finngen.fi/pheno/I9_UAP> | European | 7058 | 197,630 |
| Coronary atherosclerosis | FinnGen | <https://r5.finngen.fi/pheno/I9_CORATHER> | European | 23,363 | 187,840 |
| Varicose veins | FinnGen | <https://r5.finngen.fi/pheno/I9_VARICVE> | European | 17,027 | 190,028 |
| Gout | CKD Gen consortium | <http://ckdgen.imbi.uni-freiburg.de/> | Mixed | 13,179 | 763,813 |
|  | FinnGen | <https://r5.finngen.fi/pheno/GOUT> | European | 3576 | 203,546 |
| Fracture | GFOC plus UKBB | <http://www.gefos.org/?q=content/data-release-2018> | European | 53,184 | 373,611 |
| Fracture of forearm | FinnGen | <https://r5.finngen.fi/pheno/ST19_FRACT_FOREA> | European | 9956 | 205,768 |
| Calculus of kidney and ureter | FinnGen | https://r5.finngen.fi/pheno/N14_CALCUKIDUR | European | 4969 | 213,445 |
| Chronic kidney disease | CKD Gen consortium | <http://ckdgen.imbi.uni-freiburg.de/> | European | 41,395 | 439,303 |
|  | FinnGen | <https://r5.finngen.fi/pheno/N14_CHRONKIDNEYDIS> | European | 3902 | 212,841 |
| Endometrial cancer | Omara TA et al GWAS | MR-Base: ebi-a-GCST006464 | European | 12,906 | 108,979 |
| Leiomyoma of uterus | FinnGen | <https://r5.finngen.fi/pheno/CD2_BENIGN_LEIOMYOMA_UTERI> | European | 18,060 | 105,519 |
| Radiation-related disorders | FinnGen | <https://r5.finngen.fi/pheno/L12_RADIATIONRELATEDSKIN> | European | 5519 | 213,273 |
| Celiac disease | Trynka G et al GWAS | MR_Base: ebi-a-GCST005523 | European | 11,812 | 11,837 |
|  | Dubois PCA et al GWAS | MR_Base: ebi-a-GCST000612 | European | 4533 | 10,750 |
| Cholelithiasis | FinnGen | <https://r5.finngen.fi/pheno/K11_CHOLELITH> | European | 19,023 | 195,144 |
| Polyarteritis nodosa | FinnGen | <https://r5.finngen.fi/pheno/M13_POLNODOSA> | European | 82 | 213,145 |
| Giant cell arteritis | FinnGen | <https://r5.finngen.fi/pheno/M13_GIANTCELL> | European | 459 | 213,145 |
| Peripheral artery disease | FinnGen | <https://r5.finngen.fi/pheno/I9_PAD> | European | 7098 | 206,541 |
| Triglycerides | GLGC | MR-Base: ieu-a-302 | European | 177,861 | - |
| Total cholesterol | GLGC | MR-Base: ieu-a-301 | European | 187,365 | - |
| Low-density lipoprotein cholesterol | GLGC | MR-Base: ieu-a-300 | European | 173,082 | - |
| High-density lipoprotein cholesterol | GLGC | MR-Base: ieu-a-299 | European | 187,167 | - |
| Systolic blood pressure | ICBP | MR-Base: ieu-b-38 | European | 757,601 | - |
| Diastolic blood pressure | ICBP | MR-Base: ieu-b-39 | European | 757,601 | - |
| Uric acid | CKD Gen consortium | <http://ckdgen.imbi.uni-freiburg.de/> | European | 288,649 | - |
| Fasting glucose | The PAGE study | MR-Base: ebi-a-GCST008032 | European | 13,556 | - |

CARDIoGRAMplusC4D, Coronary ARtery DIsease Genome wide Replication and Meta-analysis (CARDIoGRAM) plus The Coronary Artery Disease (C4D) Genetics; DIAGRAM, DIAbetes Genetics Replication And Meta-analysis; GFOC, GEnetic Factors for OSteoporosis Consortium; GLGC, Global Lipids Genetics Consortium; GWAS, genome-wide association study; ICBP, International Consortium of Blood Pressure; PAGE, Population Architecture using Genomics and Epidemiology.

**Supplementary Table 4.** Characteristics of participants in the UK Biobank

| **Characteristics** | **Mean (SD)/ N (%)** |
| --- | --- |
| N | 339,197 |
| Age, mean (SD) | 56.9 (8.0) |
| Female, N (%) | 182,072 (53.7) |
| BMI, kg/m^2^, mean (SD) | 27.4 (4.8) |
| SBP mmHg, mean (SD) | 139.0 (18.7) |
| On antihypertensive medications, N (%) | 74,507 (22.0) |
| LDL cholesterol, mmol/L, mean (SD) | 3.57 (0.9) |
| On lipid-lowering medications, N (%) | 63,652 (18.8) |
| HbA1c, mmol/mol, mean (SD) | 35.95 (6.5) |
| History of diabetes mellitus, N (%) | 26,100 (7.7) |
| Current smoker, N (%) | 34,024 (10.0) |

**Supplementary Table 5.** Outcomes included in the analyses and outcomes excluded due to power (N<120)

| **Diagnostic category** | **N Phenotypes** | | | **N cases** | | |
| --- | --- | --- | --- | --- | --- | --- |
|  | **Total** | **Excluded due to power** | **Finally included** | **Median** | **Minimum** | **Maximum** |
| Circulatory system | 145 | 19 | 126 | 1747 | 124 | 96779 |
| Congenital anomalies | 52 | 19 | 33 | 470 | 543 | 1634 |
| Dermatologic | 79 | 20 | 59 | 923 | 132 | 8884 |
| Digestive | 144 | 15 | 129 | 1694 | 122 | 46601 |
| Endocrine/ Metabolic | 109 | 20 | 89 | 4348 | 122 | 47652 |
| Genitourinary | 150 | 19 | 131 | 1378 | 126 | 21598 |
| Hematopoietic | 50 | 12 | 38 | 596 | 134 | 17972 |
| Infectious disease | 52 | 17 | 35 | 641 | 127 | 12313 |
| Injuries/poisonings | 77 | 19 | 58 | 686 | 130 | 25606 |
| Mental disorders | 62 | 11 | 51 | 671 | 121 | 19366 |
| Musculoskeletal | 120 | 28 | 92 | 718 | 121 | 55332 |
| Neoplasms | 127 | 12 | 115 | 1229 | 130 | 29647 |
| Neurological | 72 | 15 | 57 | 1331 | 481 | 13230 |
| Pregnancy complications | 27 | 6 | 21 | 405 | 122 | 2401 |
| Respiratory | 71 | 7 | 64 | 1800 | 124 | 18440 |
| Sense organs | 105 | 17 | 88 | 821 | 129 | 33716 |
| Symptoms | 31 | 6 | 25 | 845 | 141 | 21745 |

**Supplementary Table 6.** Disease outcomes associated with the weighted genetic risk score of sex hormone binding globulin in phenome-wide MR analysis

| **Phecode** | **Outcome** | **Cases** | **Controls** | **Beta** | **OR** | **95% CI** | **P** |
| --- | --- | --- | --- | --- | --- | --- | --- |
| **Female and male combined** |  |  |  |  |  |  |  |
| 337.1 | Peripheral autonomic neuropathy | 252 | 321374 | -1.52 | 0.22 | 0.1, 0.49 | 2.11E-04 |
| 250.23 | Type 2 diabetes with ophthalmic manifestations | 1934 | 312261 | -1.03 | 0.36 | 0.27, 0.48 | 8.46E-12 |
| 250.2 | Type 2 diabetes | 23991 | 312261 | -0.92 | 0.40 | 0.36, 0.44 | 5.65E-88 |
| 250 | Diabetes mellitus | 24824 | 312261 | -0.88 | 0.42 | 0.38, 0.45 | 1.75E-83 |
| 401.2 | Hypertensive heart and/or renal disease | 1902 | 241393 | -0.67 | 0.51 | 0.38, 0.69 | 9.77E-06 |
| 250.4 | Abnormal glucose | 1591 | 312261 | -0.64 | 0.53 | 0.38, 0.73 | 9.60E-05 |
| 274.1 | Gout | 4486 | 333174 | -0.62 | 0.54 | 0.44, 0.66 | 5.98E-10 |
| 182 | Malignant neoplasm of uterus | 1920 | 304241 | -0.58 | 0.56 | 0.42, 0.75 | 1.16E-04 |
| 274 | Gout and other crystal arthropathies | 4998 | 333174 | -0.52 | 0.60 | 0.5, 0.72 | 3.83E-08 |
| 411.1 | Unstable angina (intermediate coronary syndrome) | 5124 | 298797 | -0.45 | 0.64 | 0.53, 0.76 | 1.11E-06 |
| 272.1 | Hyperlipidemia | 47448 | 290520 | -0.44 | 0.65 | 0.61, 0.69 | 1.09E-37 |
| 272 | Disorders of lipoid metabolism | 47652 | 290520 | -0.43 | 0.65 | 0.61, 0.69 | 1.60E-37 |
| 272.11 | Hypercholesterolemia | 43956 | 290520 | -0.43 | 0.65 | 0.61, 0.7 | 7.93E-35 |
| 594.1 | Calculus of kidney | 4153 | 329898 | -0.42 | 0.66 | 0.54, 0.8 | 3.80E-05 |
| 411.3 | Angina pectoris | 18919 | 298797 | -0.39 | 0.68 | 0.61, 0.75 | 6.18E-15 |
| 411.2 | Myocardial infarction | 13363 | 298797 | -0.33 | 0.72 | 0.64, 0.81 | 2.88E-08 |
| 411.4 | Coronary atherosclerosis | 25046 | 298797 | -0.32 | 0.73 | 0.67, 0.79 | 7.22E-13 |
| 401.1 | Essential hypertension | 96557 | 241393 | -0.31 | 0.73 | 0.7, 0.77 | 3.97E-30 |
| 401 | Hypertension | 96779 | 241393 | -0.31 | 0.73 | 0.7, 0.77 | 5.25E-30 |
| 411.8 | Other chronic ischemic heart disease, unspecified | 20560 | 298797 | -0.29 | 0.75 | 0.68, 0.82 | 1.99E-09 |
| 411 | Ischemic Heart Disease | 37856 | 298797 | -0.27 | 0.76 | 0.71, 0.82 | 2.42E-13 |
| 585.3 | Chronic renal failure [CKD] | 11321 | 311073 | -0.25 | 0.78 | 0.68, 0.88 | 6.52E-05 |
| 218 | Benign neoplasm of uterus | 10013 | 312074 | -0.25 | 0.78 | 0.68, 0.88 | 1.56E-04 |
| 585 | Renal failure | 21598 | 311073 | -0.18 | 0.84 | 0.76, 0.92 | 1.43E-04 |
| 454.1 | Varicose veins of lower extremity | 11756 | 309407 | 0.27 | 1.31 | 1.16, 1.47 | 1.39E-05 |
| 454 | Varicose veins | 12317 | 309407 | 0.28 | 1.32 | 1.17, 1.48 | 4.15E-06 |
| 803 | Fracture of upper limb | 10196 | 318888 | 0.33 | 1.39 | 1.22, 1.58 | 4.91E-07 |
| 803.2 | Fracture of radius and ulna | 7081 | 318888 | 0.44 | 1.55 | 1.33, 1.81 | 2.29E-08 |
| **Female** |  |  |  |  |  |  |  |
| 440 | Atherosclerosis | 430 | 176423 | -1.15 | 0.32 | 0.17, 0.59 | 2.69E-04 |
| 250.23 | Type 2 diabetes with ophthalmic manifestations | 699 | 171231 | -1.02 | 0.36 | 0.22, 0.59 | 4.34E-05 |
| 250.2 | Type 2 diabetes | 9401 | 171231 | -0.97 | 0.38 | 0.33, 0.44 | 1.69E-40 |
| 250 | Diabetes mellitus | 9827 | 171231 | -0.90 | 0.41 | 0.35, 0.47 | 4.56E-37 |
| 626.13 | Irregular menstrual cycle | 1670 | 153720 | -0.58 | 0.56 | 0.41, 0.77 | 2.92E-04 |
| 182 | Malignant neoplasm of uterus | 1920 | 152695 | -0.58 | 0.56 | 0.42, 0.75 | 1.16E-04 |
| 272.1 | Hyperlipidemia | 19291 | 162199 | -0.36 | 0.70 | 0.63, 0.77 | 4.37E-12 |
| 272.11 | Hypercholesterolemia | 17857 | 162199 | -0.36 | 0.70 | 0.63, 0.78 | 2.34E-11 |
| 272 | Disorders of lipoid metabolism | 19371 | 162199 | -0.35 | 0.70 | 0.64, 0.78 | 9.88E-12 |
| 401.1 | Essential hypertension | 44551 | 136931 | -0.31 | 0.73 | 0.68, 0.79 | 1.51E-15 |
| 401 | Hypertension | 44639 | 136931 | -0.31 | 0.74 | 0.68, 0.79 | 1.69E-15 |
| 411.4 | Coronary atherosclerosis | 6522 | 168277 | -0.29 | 0.75 | 0.63, 0.88 | 4.44E-04 |
| 218 | Benign neoplasm of uterus | 10013 | 161035 | -0.25 | 0.78 | 0.68, 0.88 | 1.56E-04 |
| 218.1 | Uterine leiomyoma | 9753 | 161035 | -0.25 | 0.78 | 0.68, 0.89 | 2.37E-04 |
| 454.1 | Varicose veins of lower extremity | 7526 | 165067 | 0.29 | 1.34 | 1.15, 1.55 | 1.70E-04 |
| 454 | Varicose veins | 7639 | 165067 | 0.30 | 1.35 | 1.16, 1.57 | 8.62E-05 |
| 803 | Fracture of upper limb | 7032 | 169758 | 0.40 | 1.49 | 1.27, 1.74 | 6.39E-07 |
| 803.2 | Fracture of radius and ulna | 5437 | 169758 | 0.49 | 1.64 | 1.37, 1.96 | 4.16E-08 |
| **Male** |  |  |  |  |  |  |  |
| 250.23 | Type 2 diabetes with ophthalmic manifestations | 1235 | 141030 | -1.03 | 0.36 | 0.25, 0.52 | 4.87E-08 |
| 250.2 | Type 2 diabetes | 14590 | 141030 | -0.89 | 0.41 | 0.36, 0.46 | 5.63E-50 |
| 250 | Diabetes mellitus | 14997 | 141030 | -0.87 | 0.42 | 0.37, 0.47 | 6.79E-49 |
| 274.1 | Gout | 3818 | 152492 | -0.59 | 0.55 | 0.45, 0.68 | 4.40E-08 |
| 274 | Gout and other crystal arthropathies | 4110 | 152492 | -0.54 | 0.58 | 0.47, 0.71 | 1.89E-07 |
| 411.1 | Unstable angina (intermediate coronary syndrome) | 3444 | 130520 | -0.50 | 0.60 | 0.48, 0.75 | 8.46E-06 |
| 272 | Disorders of lipoid metabolism | 28281 | 128321 | -0.49 | 0.61 | 0.56, 0.67 | 4.69E-28 |
| 272.1 | Hyperlipidemia | 28157 | 128321 | -0.49 | 0.61 | 0.56, 0.67 | 9.14E-28 |
| 594.1 | Calculus of kidney | 2767 | 150866 | -0.49 | 0.62 | 0.48, 0.79 | 1.01E-04 |
| 272.11 | Hypercholesterolemia | 26099 | 128321 | -0.48 | 0.62 | 0.56, 0.68 | 1.56E-25 |
| 411.3 | Angina pectoris | 12283 | 130520 | -0.48 | 0.62 | 0.55, 0.7 | 3.52E-14 |
| 990 | Effects radiation NOS | 3582 | 148867 | -0.46 | 0.63 | 0.51, 0.79 | 3.67E-05 |
| 585.33 | Chronic Kidney Disease, Stage III | 3417 | 141622 | -0.43 | 0.65 | 0.52, 0.81 | 1.49E-04 |
| 585.3 | Chronic renal failure [CKD] | 6041 | 141622 | -0.36 | 0.70 | 0.59, 0.83 | 3.25E-05 |
| 411.2 | Myocardial infarction | 9842 | 130520 | -0.36 | 0.70 | 0.61, 0.8 | 2.32E-07 |
| 411.4 | Coronary atherosclerosis | 18524 | 130520 | -0.33 | 0.72 | 0.65, 0.8 | 4.71E-10 |
| 411.8 | Other chronic ischemic heart disease, unspecified | 14479 | 130520 | -0.32 | 0.72 | 0.64, 0.81 | 4.14E-08 |
| 411 | Ischemic Heart Disease | 25221 | 130520 | -0.32 | 0.72 | 0.66, 0.79 | 3.17E-12 |
| 401.1 | Essential hypertension | 52006 | 104462 | -0.31 | 0.73 | 0.68, 0.79 | 2.90E-16 |
| 401 | Hypertension | 52140 | 104462 | -0.31 | 0.73 | 0.68, 0.79 | 3.38E-16 |

CI, confidence interval; OR, odds ratio.

**Supplementary Table 7.** Disease outcomes associated with the weighted genetic risk score of testosterone in phenome-wide MR analysis

| **Phecode** | **Outcome** | **Cases** | **Controls** | **Beta** | **OR** | **95% CI** | **P** |
| --- | --- | --- | --- | --- | --- | --- | --- |
| **Female and male combined** |  |  |  |  |  |  |  |
| 316.1 | Polyneuropathy due to drugs | 164 | 322863 | -2.51 | 0.08 | 0.02, 0.32 | 2.94E-04 |
| 557.1 | Celiac disease | 2185 | 274334 | -1.01 | 0.36 | 0.25, 0.53 | 1.20E-07 |
| 357 | Inflammatory and toxic neuropathy | 2326 | 335111 | -0.67 | 0.51 | 0.36, 0.74 | 3.31E-04 |
| 274.1 | Gout | 4486 | 333174 | -0.57 | 0.56 | 0.43, 0.74 | 3.02E-05 |
| 274 | Gout and other crystal arthropathies | 4998 | 333174 | -0.49 | 0.62 | 0.48, 0.79 | 1.84E-04 |
| 411.1 | Unstable angina (intermediate coronary syndrome) | 5124 | 298797 | -0.46 | 0.63 | 0.49, 0.81 | 2.85E-04 |
| 272.1 | Hyperlipidemia | 47448 | 290520 | -0.32 | 0.72 | 0.66, 0.79 | 3.80E-12 |
| 272 | Disorders of lipoid metabolism | 47652 | 290520 | -0.32 | 0.73 | 0.66, 0.79 | 5.54E-12 |
| 272.11 | Hypercholesterolemia | 43956 | 290520 | -0.31 | 0.73 | 0.67, 0.81 | 1.42E-10 |
| 411.4 | Coronary atherosclerosis | 25046 | 298797 | -0.27 | 0.76 | 0.68, 0.86 | 7.72E-06 |
| 250.2 | Type 2 diabetes | 23991 | 312261 | -0.23 | 0.80 | 0.70, 0.90 | 3.29E-04 |
| 411.8 | Other chronic ischemic heart disease, unspecified | 20560 | 298797 | -0.23 | 0.80 | 0.70, 0.91 | 7.66E-04 |
| 250 | Diabetes mellitus | 24824 | 312261 | -0.22 | 0.80 | 0.71, 0.91 | 4.28E-04 |
| 411 | Ischemic Heart Disease | 37856 | 298797 | -0.19 | 0.82 | 0.75, 0.91 | 1.48E-04 |
| 574 | Cholelithiasis and cholecystitis | 18349 | 317380 | 0.32 | 1.37 | 1.20, 1.57 | 4.58E-06 |
| 574.1 | Cholelithiasis | 16383 | 317380 | 0.33 | 1.40 | 1.21, 1.61 | 4.01E-06 |
| **Female** |  |  |  |  |  |  |  |
| 272.1 | Hyperlipidemia | 19291 | 162199 | -0.30 | 0.74 | 0.64, 0.85 | 2.33E-05 |
| 272 | Disorders of lipoid metabolism | 19371 | 162199 | -0.29 | 0.75 | 0.65, 0.86 | 3.59E-05 |
| 272.11 | Hypercholesterolemia | 17857 | 162199 | -0.28 | 0.75 | 0.65, 0.87 | 1.15E-04 |
| 574 | Cholelithiasis and cholecystitis | 12172 | 168034 | 0.35 | 1.42 | 1.20, 1.67 | 4.43E-05 |
| 574.1 | Cholelithiasis | 11015 | 168034 | 0.39 | 1.47 | 1.24, 1.75 | 1.37E-05 |
| **Male** |  |  |  |  |  |  |  |
| 557.1 | Celiac disease | 782 | 128836 | -1.51 | 0.22 | 0.12, 0.41 | 2.18E-06 |
| 411.1 | Unstable angina (intermediate coronary syndrome) | 3444 | 130520 | -0.58 | 0.56 | 0.41, 0.76 | 1.88E-04 |
| 274.1 | Gout | 3818 | 152492 | -0.56 | 0.57 | 0.43, 0.77 | 1.92E-04 |
| 274 | Gout and other crystal arthropathies | 4110 | 152492 | -0.54 | 0.58 | 0.44, 0.77 | 1.65E-04 |
| 272 | Disorders of lipoid metabolism | 28281 | 128321 | -0.34 | 0.71 | 0.63, 0.80 | 3.57E-08 |
| 272.1 | Hyperlipidemia | 28157 | 128321 | -0.34 | 0.71 | 0.63, 0.80 | 3.87E-08 |
| 272.11 | Hypercholesterolemia | 26099 | 128321 | -0.33 | 0.72 | 0.64, 0.82 | 3.05E-07 |
| 411.8 | Other chronic ischemic heart disease, unspecified | 14479 | 130520 | -0.32 | 0.72 | 0.62, 0.85 | 7.87E-05 |
| 411.4 | Coronary atherosclerosis | 18524 | 130520 | -0.29 | 0.75 | 0.65, 0.87 | 7.87E-05 |
| 411 | Ischemic Heart Disease | 25221 | 130520 | -0.25 | 0.78 | 0.69, 0.88 | 8.94E-05 |

CI, confidence interval; OR, odds ratio.

**Supplementary Table 8.** Disease outcomes associated with the weighted genetic risk score of estradiol in phenome-wide MR analysis (male only).

| **Phecode** | **Outcome** | **Cases** | **Controls** | **Beta** | **OR** | **95% CI** | **P** |
| --- | --- | --- | --- | --- | --- | --- | --- |
| **Male** |  |  |  |  |  |  |  |
| 272 | Disorders of lipoid metabolism | 28281 | 128321 | -1.99 | 0.14 | 0.07, 0.27 | 5.41E-09 |
| 272.1 | Hyperlipidemia | 28157 | 128321 | -1.96 | 0.14 | 0.07, 0.28 | 9.70E-09 |
| 272.11 | Hypercholesterolemia | 26099 | 128321 | -1.89 | 0.15 | 0.08, 0.30 | 8.31E-08 |
| 446 | Polyarteritis nodosa | 514 | 149236 | -8.01 | 0.0003 | 0.0001, 0.02 | 0.0003 |
| 443 | Peripheral vascular disease | 3281 | 149236 | -3.16 | 0.040 | 0.008, 0.24 | 0.0003 |
| 446.5 | Giant cell arteritis | 225 | 149236 | -11.81 | 0.0000 | 0.0000, 0.005 | 0.0004 |

CI, confidence interval; OR, odds ratio.

**Supplementary Table 9.** Associations of genetically predicted sex hormone-binding globulin levels with diseases and biomarkers in Mendelian randomization sensitivity analyses

| **Outcome** | **Source** | **N_SNPs** | **Cochrane’s Q** | **P_intercept_** | **Weighted median** | | **MR-Egger** | |
| --- | --- | --- | --- | --- | --- | --- | --- | --- |
|  |  |  |  |  | **OR (95% CI)** | **P** | **OR (95% CI)** | **P** |
| Hypertension | FinnGen | 406 | 1084 | 0.070 | 1.05 (0.88, 1.25) | 0.609 | 0.93 (0.76, 1.15) | 0.507 |
| Type 2 diabetes | DIAGRAM | 400 | 3886 | <0.001 | 0.75 (0.66, 0.85) | <0.001 | 0.91 (0.69, 1.19) | 0.492 |
| Type 2 diabetes | FinnGen | 406 | 1533 | <0.001 | 0.78 (0.63, 0.97) | 0.023 | 0.82 (0.62, 1.09) | 0.174 |
| Diabetic ophthalmic complications | FinnGen | 406 | 589 | 0.158 | 0.63 (0.34, 1.14) | 0.128 | 0.81 (0.47, 1.40) | 0.452 |
| Diabetic neuropathy | FinnGen | 406 | 397 | 0.140 | 0.45 (0.23, 0.88) | 0.019 | 0.74 (0.43, 1.28) | 0.285 |
| Hypertensive heart disease | FinnGen | 406 | 458 | 0.254 | 1.28 (0.81, 2.01) | 0.291 | 1.14 (0.79, 1.64) | 0.495 |
| Hypertensive renal disease | FinnGen | 406 | 441 | 0.504 | 0.67 (0.18, 2.46) | 0.550 | 0.44 (0.17, 1.16) | 0.098 |
| Coronary artery disease | CARDIoGRAMplusC4D plus UKBB | 435 | 1144 | 0.427 | 1.02 (0.90, 1.15) | 0.768 | 0.84 (0.72, 0.99) | 0.035 |
| Coronary artery disease | FinnGen | 406 | 674 | 0.337 | 0.89 (0.73, 1.09) | 0.265 | 0.90 (0.74, 1.10) | 0.304 |
| Coronary atherosclerosis | FinnGen | 406 | 676 | 0.213 | 0.84 (0.66, 1.08) | 0.174 | 0.90 (0.72, 1.13) | 0.365 |
| Myocardial infarction | CARDIoGRAMplusC4D plus UKBB | 429 | 1105 | 0.150 | 0.94 (0.81, 1.10) | 0.467 | 0.86 (0.73, 1.03) | 0.098 |
| Myocardial infarction | FinnGen | 406 | 576 | 0.264 | 0.93 (0.68, 1.27) | 0.635 | 0.91 (0.70, 1.18) | 0.472 |
| Angina pectoris | FinnGen | 406 | 644 | 0.297 | 0.93 (0.72, 1.19) | 0.546 | 0.86 (0.67, 1.09) | 0.207 |
| Unstable angina | FinnGen | 406 | 530 | 0.139 | 1.08 (0.79, 1.49) | 0.623 | 0.92 (0.67, 1.26) | 0.587 |
| Varicose veins | FinnGen | 406 | 823 | 0.025 | 1.37 (1.07, 1.75) | 0.014 | 1.06 (0.81, 1.38) | 0.662 |
| Gout | CKD Gen consortium | 395 | 997 | 0.081 | 1.01 (0.79, 1.28) | 0.959 | 0.65 (0.48, 0.87) | 0.004 |
| Gout | FinnGen | 406 | 530 | 0.544 | 0.76 (0.47, 1.23) | 0.256 | 0.61 (0.41, 0.91) | 0.015 |
| Fracture | GFOC plus UKBB | 400 | 552 | 0.030 | 0.99 (0.88, 1.12) | 0.893 | 1.02 (0.92, 1.14) | 0.661 |
| Fracture of forearm | FinnGen | 406 | 547 | 0.537 | 1.13 (0.89, 1.44) | 0.326 | 1.23 (0.97, 1.57) | 0.092 |
| Calculus of kidney and ureter | FinnGen | 406 | 582 | 0.294 | 0.91 (0.61, 1.35) | 0.631 | 0.90 (0.64, 1.28) | 0.567 |
| Chronic kidney disease | CKD Gen consortium | 414 | 717 | 0.298 | 0.87 (0.73, 1.04) | 0.137 | 0.98 (0.82, 1.17) | 0.794 |
| Chronic kidney disease | FinnGen | 406 | 387 | 0.386 | 0.96 (0.61, 1.51) | 0.872 | 0.93 (0.67, 1.28) | 0.643 |
| Endometrial cancer | Omara TA et al GWAS | 447 | 624 | 0.443 | 0.66 (0.49, 0.90) | 0.008 | 0.68 (0.53, 0.88) | 0.003 |
| Leiomyoma of uterus | FinnGen | 406 | 634 | 0.593 | 0.90 (0.71, 1.15) | 0.406 | 0.79 (0.63, 1.00) | 0.050 |
| Radiation-related disorders | FinnGen | 406 | 515 | 0.206 | 0.98 (0.71, 1.36) | 0.897 | 0.09 (0.65, 1.23) | 0.505 |
|  |  |  |  |  | **Change (95% CI)** | ***P*** | **Change (95% CI)** | ***P*** |
| Total cholesterol | GLGC | 302 | 1581 | 0.406 | -0.06 (-0.17, 0.05) | 0.304 | -0.16 (-0.34, 0.02) | 0.091 |
| Low-density lipoprotein cholesterol | GLGC | 301 | 1311 | 0.598 | -0.12 (-0.23, 0.00) | 0.044 | -0.10 (-0.27, 0.07) | 0.234 |
| High-density lipoprotein cholesterol | GLGC | 302 | 1536 | 0.008 | 0.20 (0.10, 0.30) | <0.001 | 0.25 (0.09, 0.42) | 0.003 |
| Triglycerides | GLGC | 301 | 1995 | 0.332 | -0.22 (-0.33, -0.12) | <0.001 | -0.54 (-0.73, -0.35) | <0.001 |
| Systolic blood pressure | ICBP | 410 | 3867 | <0.001 | -1.11 (-1.91, -0.31) | 0.007 | -0.61 (-1.93, 0.71) | 0.365 |
| Diastolic blood pressure | ICBP | 412 | 4149 | 0.008 | 0.20 (-0.14, 0.54) | 0.244 | -0.43 (-1.23, 0.36) | 0.286 |
| Uric acid | CKD Gen consortium | 392 | 1759 | 0.028 | -0.01 (-0.09, 0.06) | 0.781 | -0.11 (-0.23, 0.01) | 0.067 |
| Fasting glucose | The PAGE study | 448 | 694 | 0.207 | -0.11 (-0.29, 0.08) | 0.253 | 0.01 (-0.16, 0.18) | 0.916 |

CARDIoGRAMplusC4D, Coronary ARtery DIsease Genome wide Replication and Meta-analysis (CARDIoGRAM) plus The Coronary Artery Disease (C4D) Genetics; CI, confidence interval; DIAGRAM, DIAbetes Genetics Replication And Meta-analysis; GFOC, GEnetic Factors for OSteoporosis Consortium; GLGC, Global Lipids Genetics Consortium; GWAS, genome-wide association study; ICBP, International Consortium of Blood Pressure; N-SNPs, number of used single nucleotide polymorphism; OR, odds ratio; PAGE, Population Architecture using Genomics and Epidemiology.

P_intercept_ is p value for intercept in MR-Egger regression.

**Supplementary Table 10.** Associations of genetically predicted testosterone levels with diseases and biomarkers in Mendelian randomization sensitivity analyses

| **Outcome** | **Source** | **N_SNPs** | **Cochrane’s Q** | **P_intercept_** | **Weighted median** | | **MR-Egger** | |
| --- | --- | --- | --- | --- | --- | --- | --- | --- |
|  |  |  |  |  | **OR (95% CI)** | **P** | **OR (95% CI)** | **P** |
| Type 2 diabetes | DIAGRAM | 154 | 1133 | 0.001 | 0.92 (0.75, 1.12) | 0.413 | 1.34 (0.91, 1.98) | 0.134 |
| Type 2 diabetes | FinnGen | 154 | 464 | 0.058 | 0.95 (0.71, 1.27) | 0.707 | 1.16 (0.75, 1.78) | 0.507 |
| Coronary artery disease | CARDIoGRAMplusC4D plus UKBB | 165 | 549 | 0.381 | 1.03 (0.87, 1.22) | 0.725 | 0.92 (0.69, 1.23) | 0.577 |
| Coronary artery disease | FinnGen | 154 | 284 | 0.552 | 0.88 (0.67, 1.17) | 0.390 | 0.85 (0.60, 1.20) | 0.348 |
| Coronary atherosclerosis | FinnGen | 154 | 281 | 0.253 | 0.85 (0.63, 1.16) | 0.319 | 0.91 (0.61, 1.34) | 0.624 |
| Angina pectoris | FinnGen | 154 | 267 | 0.385 | 0.98 (0.69, 1.38) | 0.888 | 0.85 (0.56, 1.30) | 0.460 |
| Unstable angina | FinnGen | 154 | 214 | 0.569 | 1.15 (0.69, 1.90) | 0.599 | 0.93 (0.54, 1.60) | 0.791 |
| Gout | CKD Gen consortium | 153 | 742 | 0.292 | 1.03 (0.76, 1.39) | 0.865 | 0.77 (0.40, 1.49) | 0.439 |
| Gout | FinnGen | 154 | 272 | 0.792 | 0.69 (0.40, 1.19) | 0.180 | 0.83 (0.38, 1.80) | 0.636 |
| Celiac disease | Trynka G et al GWAS | 13 | 21 | 0.180 | 0.33 (0.11, 1.00) | 0.050 | 0.12 (0.01, 1.27) | 0.105 |
| Celiac disease | Dubois PCA et al GWAS | 76 | 86 | 0.882 | 0.50 (0.21, 1.21) | 0.125 | 0.64 (0.18, 2.32) | 0.499 |
| Cholelithiasis | FinnGen | 154 | 440 | 0.087 | 1.23 (0.93, 1.64) | 0.143 | 1.84 (1.15, 2.95) | 0.011 |
|  |  |  |  |  | **Change (95% CI)** | **P** | **Change (95% CI)** | **P** |
| Total cholesterol | GLGC | 119 | 543 | 0.270 | -0.04 (-0.19, 0.11) | 0.564 | -0.4 (-0.75, -0.05) | 0.028 |
| Low-density lipoprotein cholesterol | GLGC | 119 | 387 | 0.913 | -0.01 (-0.16, 0.14) | 0.914 | -0.09 (-0.40, 0.22) | 0.571 |
| High-density lipoprotein cholesterol | GLGC | 119 | 492 | 0.873 | 0.16 (0.02, 0.31) | 0.028 | 0.19 (-0.13, 0.52) | 0.241 |
| Triglycerides | GLGC | 119 | 1533 | 0.002 | -0.25 (-0.38, -0.11) | <0.001 | -1.47 (-2.00, -0.94) | <0.001 |
| Uric acid | CKD Gen consortium | 154 | 1496 | 0.228 | 0.09 (-0.02, 0.19) | 0.105 | 0.02 (-0.27, 0.30) | 0.909 |

CARDIoGRAMplusC4D, Coronary ARtery DIsease Genome wide Replication and Meta-analysis (CARDIoGRAM) plus The Coronary Artery Disease (C4D) Genetics; CI, confidence interval; DIAGRAM, DIAbetes Genetics Replication And Meta-analysis; GFOC, GEnetic Factors for OSteoporosis Consortium; GLGC, Global Lipids Genetics Consortium; GWAS, genome-wide association study; N-SNPs, number of used single nucleotide polymorphism; OR, odds ratio.

P_intercept_ is p value for intercept in MR-Egger regression.

**Supplementary Table 11.** Associations of genetically predicted estradiol levels with diseases and biomarkers in Mendelian randomization analyses

| **Sources** | **Cases** | **Controls** | **N_SNPs** | **Cochrane’s Q** | **P_intercept_** | **IVW-random effects** | | | **Weighted median** | | | **MR-Egger** | | |
| --- | --- | --- | --- | --- | --- | --- | --- | --- | --- | --- | --- | --- | --- | --- |
|  |  |  |  |  |  | **Beta** | **SE** | **p** | **Beta** | **SE** | **p** | **Beta** | **SE** | **p** |
| Total cholesterol | | |  |  |  |  |  |  |  |  |  |  |  |  |
| GLGC | 187,365 | - | 5 | 158 | 0.142 | -1.850 | 1.600 | 0.247 | -0.297 | 0.307 | 0.333 | 3.399 | 2.913 | 0.328 |
| Low-density lipoprotein cholesterol | | |  |  |  |  |  |  |  |  |  |  |  |  |
| GLGC | 173,082 | - | 5 | 25 | 0.048 | -0.921 | 0.655 | 0.159 | -0.435 | 0.329 | 0.186 | 1.594 | 0.854 | 0.159 |
| High-density lipoprotein cholesterol | | | |  |  |  |  |  |  |  |  |  |  |  |
| GLGC | 187,167 | - | 5 | 26 | 0.106 | 0.250 | 0.609 | 0.681 | -0.262 | 0.315 | 0.405 | -1.866 | 1.017 | 0.164 |
| Triglycerides | | | |  |  |  |  |  |  |  |  |  |  |  |
| GLGC | 177,861 | - | 5 | 914 | 0.154 | -3.587 | 3.515 | 0.308 | -0.290 | 0.291 | 0.320 | 7.692 | 6.544 | 0.325 |
| FinnGen |  |  |  |  |  |  |  |  |  |  |  |  |  |  |
| Polyarteritis nodosa | 514 | 149236 | 11 | 17 | 0.689 | -0.455 | 8.048 | 0.955 | -6.409 | 7.977 | 0.422 | -8.075 | 20.249 | 0.699 |
| Peripheral vascular disease | 3281 | 149236 | 11 | 16 | 0.146 | -0.708 | 0.931 | 0.447 | -0.019 | 1.005 | 0.985 | 2.317 | 2.087 | 0.296 |
| Giant cell arteritis | 225 | 149236 | 11 | 9 | 0.310 | -1.459 | 2.641 | 0.581 | -3.044 | 3.674 | 0.407 | -8.010 | 6.694 | 0.262 |

GLGC, Global Lipids Genetics Consortium; IVW, inverse variance weighted; N-SNPs, number of used single nucleotide polymorphism; OR, odds ratio; SE, standard error.

P_intercept_ is p value for intercept in MR-Egger regression.

**Supplementary Table 12.** Mediation effects in two-sample MR analyses for genetically predicted sex hormone-binding globulin levels.

| **Outcome** | **Source** | **Cases** | **Controls** | **Mediation by lipids** | **Mediation by BPs** | **Mediation by urate** |
| --- | --- | --- | --- | --- | --- | --- |
| Hypertension | FinnGen | 55917 | 162837 | 95.2% | 72.3% | 39.3% |
| Type 2 diabetes | DIAGRAM | 74124 | 824006 | 41.8% | 33.0% | 11.0% |
|  | FinnGen | 29166 | 183185 | 37.9% | 35.6% | 5.7% |
|  | Meta-analysis |  |  | 39.6% | 35.2% | 11.0% |
| Diabetic ophthalmic complications | FinnGen | 2119 | 183185 | 30.2% | 35.7% | 13.5% |
| Diabetic neuropathy | FinnGen | 1415 | 162201 | 8.9% | 0.0% | 3.1% |
| Coronary artery disease | CARDIoGRAMplusC4D plus UKBB | 122733 | 424528 | 81.7% | 42.7% | 32.4% |
|  | FinnGen | 30952 | 187840 | 100.0% | 64.5% | 52.2% |
|  | Meta-analysis |  |  | 89.8% | 52.5% | 35.6% |
| Coronary atherosclerosis | FinnGen | 23363 | 187840 | 100.0% | 60.4% | 44.7% |
| Myocardial infarction | CARDIoGRAMplusC4D plus UKBB | 61505 | 577716 | 70.8% | 39.3% | 29.8% |
|  | FinnGen | 11622 | 187840 | 90.4% | 75.7% | 60.4% |
|  | Meta-analysis |  |  | 78.2% | 45.8% | 36.0% |
| Angina pectoris | FinnGen | 18168 | 187840 | 96.0% | 53.1% | 34.6% |
| Unstable angina | FinnGen | 7058 | 197630 | 100.0% | 51.1% | 37.8% |
| Varicose veins | FinnGen | 17027 | 190028 | 54.1% | 8.0% | 5.2% |
| Gout | CKD Gen consortium | 13179 | 763813 | 54.7% | 24.7% | 52.6% |
|  | FinnGen | 3576 | 203546 | 38.0% | 21.4% | 47.4% |
|  | Meta-analysis |  |  | 49.6% | 20.9% | 49.6% |
| Fracture | GFOC plus UKBB | 53184 | 373611 | 48.6% | 32.1% | 15.9% |
| Fracture of forearm | FinnGen | 9956 | 205768 | 35.6% | 29.4% | 14.4% |
| Calculus of kidney and ureter | FinnGen | 4969 | 213445 | 62.0% | 15.2% | 20.1% |
| Chronic kidney disease | CKD Gen consortium | 41395 | 439303 | 0.0% | 11.7% | 23.1% |
|  | FinnGen | 3902 | 212841 | 43.5% | 61.1% | 12.8% |
|  | Meta-analysis |  |  | 19.1% | 19.1% | 28.4% |
| Endometrial cancer | Omara TA et al GWAS | 12906 | 108979 | 8.8% | 8.6% | 4.3% |
| Leiomyoma of uterus | FinnGen | 18060 | 105519 | 78.1% | 25.3% | 31.4% |

**Supplementary Table 13.** Associations of genetically predicted sex hormone-binding globulin levels with diseases in univariable and multivariable Mendelian randomization analysis

| **Outcome** | **Source** | **Cases** | **Controls** | **Univariable IVW analysis** | | **MVMR adjusted for urate** | |
| --- | --- | --- | --- | --- | --- | --- | --- |
|  |  |  |  | **OR (95% CI)** | ***P*** | **OR (95% CI)** | ***P*** |
| Hypertension | FinnGen | 55917 | 162837 | 0.81 (0.70, 0.93) | 0.002 | 0.88 (0.76, 1.01) | 0.066 |
| Hypertensive heart disease | FinnGen | 3938 | 162837 | 0.97 (0.76, 1.24) | 0.798 | 1.01 (0.78, 1.30) | 0.947 |
| Hypertensive renal disease | FinnGen | 468 | 162837 | 0.57 (0.30, 1.07) | 0.082 | 0.59 (0.30, 1.18) | 0.138 |
| Type 2 diabetes | DIAGRAM | 74124 | 824006 | 0.49 (0.40, 0.59) | <0.001 | 0.53 (0.43, 0.64) | <0.001 |
|  | FinnGen | 29166 | 183185 | 0.50 (0.41, 0.61) | <0.001 | 0.52 (0.42, 0.65) | <0.001 |
|  | Meta-analysis |  |  | 0.49 (0.43, 0.57) | <0.001 | 0.53 (0.46, 0.61) | <0.001 |
| Diabetic ophthalmic complications | FinnGen | 2119 | 183185 | 0.60 (0.42, 0.87) | 0.007 | 0.56 (0.38, 0.82) | 0.003 |
| Diabetic neuropathy | FinnGen | 1415 | 162201 | 0.55 (0.38, 0.78) | <0.001 | 0.54 (0.37, 0.80) | 0.002 |
| Coronary artery disease | CARDIoGRAMplusC4D plus UKBB | 122733 | 424528 | 0.80 (0.72, 0.89) | <0.001 | 0.86 (0.77, 0.95) | 0.005 |
|  | FinnGen | 30952 | 187840 | 0.84 (0.73, 0.96) | 0.009 | 0.92 (0.80, 1.05) | 0.207 |
|  | Meta-analysis |  |  | 0.82 (0.75, 0.88) | <0.001 | 0.88 (0.81, 0.96) | 0.003 |
| Coronary atherosclerosis | FinnGen | 23363 | 187840 | 0.81 (0.70, 0.94) | 0.006 | 0.89 (0.76, 1.04) | 0.157 |
| Myocardial infarction | CARDIoGRAMplusC4D plus UKBB | 61505 | 577716 | 0.78 (0.70, 0.88) | <0.001 | 0.84 (0.74, 0.94) | 0.002 |
|  | FinnGen | 11622 | 187840 | 0.81 (0.69, 0.97) | 0.019 | 0.92 (0.76, 1.10) | 0.338 |
|  | Meta-analysis |  |  | 0.79 (0.72, 0.87) | <0.001 | 0.86 (0.78, 0.95) | 0.002 |
| Angina pectoris | FinnGen | 18168 | 187840 | 0.78 (0.66, 0.91) | 0.002 | 0.85 (0.73, 1.00) | 0.063 |
| Unstable angina | FinnGen | 7058 | 197630 | 0.77 (0.62, 0.95) | 0.013 | 0.85 (0.68, 1.07) | 0.160 |
| Varicose veins | FinnGen | 17027 | 190028 | 1.33 (1.11, 1.59) | 0.002 | 1.35 (1.12, 1.63) | 0.002 |
| Gout | CKD Gen consortium | 13179 | 763813 | 0.53 (0.44, 0.65) | <0.001 | 0.74 (0.63, 0.87) | <0.001 |
|  | FinnGen | 3576 | 203546 | 0.67 (0.51, 0.87) | 0.003 | 0.81 (0.62, 1.06) | 0.128 |
|  | Meta-analysis |  |  | 0.58 (0.49, 0.67) | <0.001 | 0.76 (0.66, 0.87) | <0.001 |
| Fracture | GFOC plus UKBB | 53184 | 373611 | 1.12 (1.04, 1.21) | 0.002 | 1.10 (1.02, 1.18) | 0.013 |
| Fracture of forearm | FinnGen | 9956 | 205768 | 1.31 (1.11, 1.54) | 0.001 | 1.26 (1.06, 1.50) | 0.007 |
| Calculus of kidney and ureter | FinnGen | 4969 | 213445 | 0.78 (0.62, 0.99) | 0.041 | 0.82 (0.63, 1.05) | 0.110 |
| Chronic kidney disease | CKD Gen consortium | 41395 | 439303 | 0.91 (0.81, 1.02) | 0.111 | 0.93 (0.83, 1.05) | 0.247 |
|  | FinnGen | 3902 | 212841 | 0.83 (0.67, 1.03) | 0.087 | 0.85 (0.67, 1.08) | 0.184 |
|  | Meta-analysis |  |  | 0.89 (0.80, 0.99) | 0.026 | 0.92 (0.82, 1.02) | 0.101 |
| Endometrial cancer | Omara TA et al GWAS | 12906 | 108979 | 0.73 (0.62, 0.86) | <0.001 | 0.74 (0.62, 0.88) | 0.001 |
| Leiomyoma of uterus | FinnGen | 18060 | 105519 | 0.83 (0.71, 0.97) | 0.018 | 0.88 (0.75, 1.04) | 0.126 |
| Radiation-related disorders | FinnGen | 5519 | 213273 | 1.05 (0.85, 1.29) | 0.675 | 1.00 (0.80, 1.25) | 0.987 |

CARDIoGRAMplusC4D, Coronary ARtery DIsease Genome wide Replication and Meta-analysis (CARDIoGRAM) plus The Coronary Artery Disease (C4D) Genetics; CI, confidence interval; DIAGRAM, DIAbetes Genetics Replication And Meta-analysis; GFOC, GEnetic Factors for OSteoporosis Consortium; GWAS, genome-wide association study; IVW, inverse variance weighted; MVMR, multivariable Mendelian randomization; OR, odds ratio.

**Supplementary Table 14.** Mediation effects in two-sample MR analyses for genetically predicted testosterone levels.

| **Outcome** | **Source** | **Cases** | **Controls** | **Mediation by lipids** |
| --- | --- | --- | --- | --- |
| Type 2 diabetes | DIAGRAM | 74124 | 824006 | 0.0% |
|  | FinnGen | 29166 | 183185 | 0.0% |
|  | Meta-analysis |  |  | 0.0% |
| Coronary artery disease | CARDIoGRAMplusC4D plus UKBB | 122733 | 424528 | 70.1% |
|  | FinnGen | 30952 | 187840 | 43.8% |
|  | Meta-analysis |  |  | 56.3% |
| Coronary atherosclerosis | FinnGen | 23363 | 187840 | 32.2% |
| Angina pectoris | FinnGen | 18168 | 187840 | 38.7% |
| Gout | CKD Gen consortium | 13179 | 763813 | 100.0% |
|  | FinnGen | 3576 | 203546 | 100.0% |
|  | Meta-analysis |  |  | 100.0% |
| Celiac disease | Trynka G et al GWAS | 11812 | 11837 | 36.1% |
|  | Dubois PCA et al GWAS | 4533 | 10750 | 5.9% |
|  | Meta-analysis |  |  | 12.2% |
| Cholelithiasis | FinnGen | 19023 | 195144 | 52.7% |


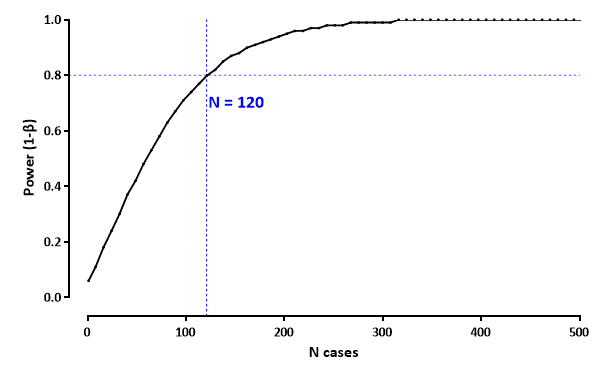


**Supplementary Figure 1.** Power calculations for the clinical endpoints (OR>2 or OR <0.5 at the significance level of α=0.05 and assuming phenotypic variance at 6.9% (corresponding to the variance explained by genetic variants associated with SHBG levels))


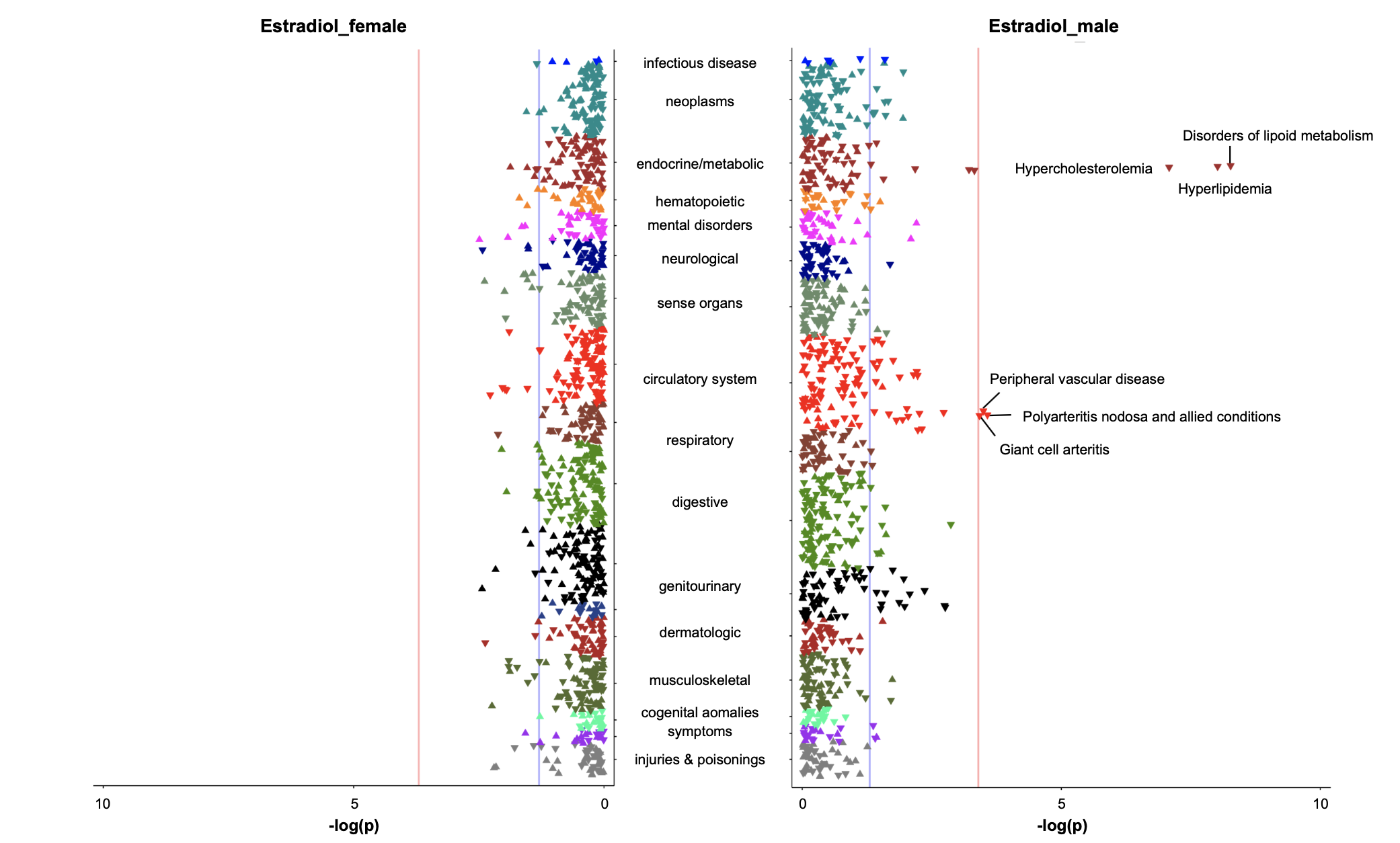


**Supplementary Figure 2**. Associations of genetically predicted estradiol levels with clinical outcomes and biomarkers in the phenome-wide association analysis in male in the UK Biobank. The y-axis corresponds to the logarithms of the *p* values derived from the phenome-wide association analyses. The red lines correspond to the statistical significance level (false discovery rate<0.05). Associations surviving the significance criteria are labeled by name. Associations that are found in female and male combined are labeled in black. The triangle facing up represents a positive association, otherwise an inverse association.
